# Meta-Analysis and Experimental Validation Identified FREM2 and SPRY1 as New Glioblastoma Marker Candidates

**DOI:** 10.3390/ijms19051369

**Published:** 2018-05-04

**Authors:** Marko Vidak, Ivana Jovcevska, Neja Samec, Alja Zottel, Mirjana Liovic, Damjana Rozman, Saso Dzeroski, Peter Juvan, Radovan Komel

**Affiliations:** 1 Faculty of Medicine, Medical Centre for Molecular Biology, University of Ljubljana, Ljubljana SI-1000, Slovenia; marko.vidak@mf.uni-lj.si (M.V.); ivana.jovcevska@mf.uni-lj.si (I.J.); neja.samec@mf.uni-lj.si (N.S.); alja.zottel@mf.uni-lj.si(A.Z.); mirjana.liovic@mf.uni-lj.si (M.L.); 2 Faculty of Medicine, Centre for Functional Genomics and Bio-Chips, University of Ljubljana, Ljubljana SI-1000, Slovenia; damjana.rozman@mf.uni-lj.si; 3 Department of Knowledge Technologies, Jozef Stefan Institute, Ljubljana SI-1000, Slovenia; saso.dzeroski@ijs.si

**Keywords:** glioblastoma, glioblastoma stem cells, biomarkers, data repositories, meta-analysis, cell surface, experimental validation, FREM2, SPRY1

## Abstract

Glioblastoma (GB) is the most aggressive brain malignancy. Although some potential glioblastoma biomarkers have already been identified, there is a lack of cell membrane-bound biomarkers capable of distinguishing brain tissue from glioblastoma and/or glioblastoma stem cells (GSC), which are responsible for the rapid post-operative tumor reoccurrence. In order to find new GB/GSC marker candidates that would be cell surface proteins (CSP), we have performed meta-analysis of genome-scale mRNA expression data from three data repositories (GEO, ArrayExpress and GLIOMASdb). The search yielded ten appropriate datasets, and three (GSE4290/GDS1962, GSE23806/GDS3885, and GLIOMASdb) were used for selection of new GB/GSC marker candidates, while the other seven (GSE4412/GDS1975, GSE4412/GDS1976, E-GEOD-52009, E-GEOD-68848, E-GEOD-16011, E-GEOD-4536, and E-GEOD-74571) were used for bioinformatic validation. The selection identified four new CSP-encoding candidate genes—*CD276*, *FREM2*, *SPRY1*, and *SLC47A1*—and the bioinformatic validation confirmed these findings. A review of the literature revealed that *CD276* is not a novel candidate, while *SLC47A1* had lower validation test scores than the other new candidates and was therefore not considered for experimental validation. This validation revealed that the expression of FREM2—but not SPRY1—is higher in glioblastoma cell lines when compared to non-malignant astrocytes. In addition, *FREM2* gene and protein expression levels are higher in GB stem-like cell lines than in conventional glioblastoma cell lines. FREM2 is thus proposed as a novel GB biomarker and a putative biomarker of glioblastoma stem cells. Both FREM2 and SPRY1 are expressed on the surface of the GB cells, while SPRY1 alone was found overexpressed in the cytosol of non-malignant astrocytes.

## 1. Introduction

Glioma is the cancer of the brain connective tissue. Histological characteristics of tumor cells are used for the classification of gliomas in several types, the most common of them being astrocytomas and oligodendrogliomas. Furthermore, gliomas are graded according to their malignancy in four grades, where grade I is of the least and grade IV of the most severe phenotype. The most aggressive and lethal form of glioma is grade IV astrocytoma, also known as glioblastoma (GB) [1]. Patients with this disease who receive the best currently available treatment—i.e., surgical tumor resection combined with radiation and adjuvant chemotherapy—survive on average 15 months after being diagnosed [2]. Resections usually fail to remove the most malignant cells, which are inconveniently located at tumor edges in close proximity to tumor-supplying blood vessels [3]. These cells exhibit stem-like properties such as partial pluripotency, lack of differentiation, and self-renewal ability. Moreover, they have pronounced migratory and invasive potential that enables their diffuse spread into brain regions essential for survival of the patient. Thus, they are known as glioblastoma stem cells or glioblastoma stem-cell like cells (GSC) and are believed to be the reason for frequently occurring post-operative tumor relapses [4,5].

Genes and their corresponding proteins with elevated expression in GB are referred to as GB markers. Some of them are also over-expressed in GSC when compared to non-stem glioblastoma cells and are thus referred to as GSC markers. Several GSC markers have already been proposed, but so far, none have been conclusively validated [6,7], so these proposed markers should be referred to as GSC marker candidates. These candidates have been most recently reviewed by Glaser et al. [8].

Although genes/proteins with decreased expression in GB/GSC could be also relevant for diagnosis and prognosis, the search for new markers has been focused on genes/proteins with elevated expression in GB/GSC. Any such candidate should have significantly higher expression levels in glioblastoma when compared to, respectively, normal brain tissue, low-grade gliomas of all histological types, and oligodendrogliomas of all grades, since glioblastoma is histologically an astrocytic tumor. Within the tumor, the GSC marker candidate is supposed to be significantly over-expressed in GSC when compared to ordinary tumor cells.

Proteins that have elevated expression in GSC and are localized at the cell surface are especially valuable as potential therapeutic targets since they are more easily accessible to be targeted by various drugs, including advanced biological drugs such as antibodies and their conjugates. The most promising cell-surface marker candidates reported so far are CD133 (PROM1) [9], CXCR4 [10], MELK [11], PTCH1 [12], CD9 [13,14], and CD44 [15]. However, the expression of these markers is not exclusive to GSC, since they are also expressed in other stem cell types including neural stem cells (NSC).

## 2. Results and Discussion

We performed meta-analysis of genome-scale mRNA expression data from three unrelated public repositories. Four genes were identified as potential new CSP-encoding glioblastoma markers. After bioinformatic validation, two of them were selected for experimental validation and one of them was confirmed as a potential novel cell-surface glioblastoma biomarker. Its expression was also experimentally confirmed in GSC.

### 2.1. Selection of Glioblastoma Datasets

The repositories Gene Expression Omnibus (GEO) [16], ArrayExpress [17], and GLIOMASdb [18] were browsed in order to obtain datasets appropriate for selection or validation of new GB/GSC marker candidates (see Table 1). The single GLIOMASdb dataset complied with the requirements for appropriate datasets (see Materials and Methods). Search in GEO (query “glioblastoma”, filters “homo sapiens” and “DataSets”) yielded four appropriate datasets: GSE4290/GDS1962, GSE23806/GDS3885, GSE4412/GDS1975, and GSE4412/GDS1976. In ArrayExpress, the query »glioblastoma, grade« (with filters »homo sapiens«, »RNA assay«, and »array assay«) yielded three appropriate datasets (E-GEOD-52009, E-GEOD-68848, and E-GEOD-16011), the query »glioblastoma, stem cells« (same filters) yielded two datasets (E-GEOD-4536 and E-GEOD-74571), while the last query »glioblastoma, neurospheres« (same filter) produced no appropriate results.

The best of the appropriate datasets—i.e., the datasets that contained curated data and/or permitted multiple comparisons relevant for selection of new GB/GSC marker candidates (see Table 1)—were chosen to be used for selection of new marker candidates. These datasets were the GEO datasets GSE4290/GDS1962 and GSE23806/GDS3885, as well as the single GLIOMASdb dataset (see Table 2). The rest of the appropriate datasets (Table 2) were used for bioinformatic validation of novel GB/GSC candidates that were identified in the selection stage.

### 2.2. Selection of New Glioblastoma Marker Candidates

We conducted eight pairwise comparisons between groups of samples from the selection datasets (i.e., GSE4290/GDS1962, GSE23806/GDS3885, and GLIOMASdb). The aim of these selection tests was to identify new GB/GSC marker candidates. Similarly, 13 pairwise comparisons were conducted for bioinformatic validation of new GB/GSC marker candidates between groups of samples from the seven independent validation datasets (i.e., GSE4412/GDS1975, GSE4412/GDS1976, E-GEOD-52009, E-GEOD-68848, E-GEOD-16011, E-GEOD-4536, and E-GEOD-74571). Additional data about the tests—e.g., the size and composition of the compared groups—are presented in Table 3. Detailed test results for each test are available in the Tables S1−S22 (see Supplementary Materials), while the Figures S1−S20 contain sample codes and boxplot distributions of sample values.

Table 4 lists the top ten genes according to the combined results of the eight tests of the selection stage (Table 3). The entire list is available in the Table S23. The selection of genes was based on false discovery rate (FDR)-adjusted *p* values (cut-off value *p* < 0.001), and on log2 of the ratio of average expression levels in the two compared groups (log change or lfc; cut-off value lfc > 1). Three of the top ten genes, including one CSP gene (CD276), met the criteria of all eight tests. Furthermore, seven other genes passed 7/8 tests, and three of them are CSP genes: *SPRY1*, *FREM2*, and *SLC47A1*. All these genes scored both *p* < 0.05 and lfc > 0 in the single failed test (where they had either *p* > 0.001 or lfc < 1), indicating that their expression was at least slightly elevated (lfc > 0) in the first test group and their *p* value was within conventionally used significance limits (*p* < 0.05). *SPRY1, FREM2*, and *SLC47A1* were thus included along *CD276* in further analysis.

### 2.3. Assessment of the Effectiveness of Selection Tests

Six established candidate genes coding for cell surface proteins (*CD133*—also known as *PROM1*, *CXCR4*, *MELK*, *PTCH1*, *CD9*, and *CD44*) were used as a benchmark for assessing the effectiveness of selection tests that identified *SPRY1*, *FREM2*, *SLC47A1*, and *CD276* as new glioblastoma marker candidates. Housekeeping genes cytochrome c1 (*CYC1*), hypoxanthine phosphoribosyltransferase 1 (*HPRT1*), H3 histone family 3A (*H3F3A*), ribosomal protein lateral stalk subunit P0 (*RPLP0*), and endoplasmatic reticulum membrane protein complex subunit 7 (*EMC7*) were used as negative control. A points-based system (described in detail in Materials and Methods) was applied in order to indicate different levels of significantly elevated gene expression. A scale from four to zero points was used—four points for the most significantly elevated expression, zero points for non-significant expression differences or genes with decreased expression. Average points per test (APPT) was used as a performance measure. 

Results of our selection tests (Table 5) show that all six established candidates scored three points or more in a majority of the tests and that all the established candidates (except *PTCH1*) scored on average more than two points in each test. These results show that our selection tests were able to expose the elevated expression of the established candidates. On the other hand, four of five housekeeping genes had APPT less than one and only *HPRT1*—purportedly universally reliable as negative control in cancer-related transcriptome analysis [23]—had APPT equal to one.

### 2.4. Validation Tests with the Data from Other Datasets

The comparisons used in selection tests that identified four new glioblastoma marker candidates were recreated with data from seven other datasets (Table 2), resulting in 13 additional validation tests (Table 3) of the same type as selection tests. The points-based system—described in Materials and Methods—was used to evaluate different levels of elevated expression. The established candidates and housekeeping genes from the previous section were also used here with the same purpose. Results of these validation tests are presented in Table 6.

*FREM2* had the highest point average (3.250) among all 15 featured genes, but it also had the highest number of missing results (5/13). In the second place among all twelve genes, *CD276* had only slightly lower point average (3.167) than *FREM2* and only one missing value. In fact, all four new candidates outperformed each of the established candidates. The worst among the established candidates was *PTCH1*, whose APPT (0.615) was lower than APPT of each control gene except *EMC7* (0.545). Two of the housekeeping genes—*H3F3A* and *RPLP0*—had APPT equal to or slightly above 1, but their APPT was still notably lower than that of *CD133* (1.917), which was after the outlier *PTCH1* the second worst-placed established candidate. All new candidates and all established candidates, except *PTCH1*, had point averages generally similar to those in the selection tests. This proves that the results of selection tests were not a consequence of the particular dataset choice. 

### 2.5. Novelty Assessment of the Identified Marker Candidates

A brief review of relevant literature (Table 4) revealed that *CD276* had already been linked to carcinogenesis of glioblastoma and other cancers [19,24]. However, for the other three candidates (*FREM2*, *SPRY1*, and *SLC47A1*) no direct association with glioblastoma has been reported so far. Since *SLC47A1* had the worst performance among these three genes in the previous stage (Table 6), it was omitted from experimental validation, which therefore included *FREM2* and *SPRY1*. Their already known association with various diseases is discussed below. 

#### 2.5.1. FREM2—Expression Patterns and Association with Disease

FREM2 is a protein localized mostly in various epithelia or in the extracellular matrix, and is widely expressed in embryonic tissues. Smaller fractions of FREM2 are also present in the cytosol and endoplasmic reticulum, which is localized close to the nuclear membrane and is the place of protein synthesis. FREM2 mRNA has been localized, among other tissues, in the embryonic neural stem cells (NSC) that are predecessors of some brain and spinal cord regions [25]. However, there are no reports of FREM2 detection in NSC in the adult brain. Biological processes in which FREM2 is involved include epithelial formation, cell adhesion and communication, and development of heart and inner ear. *FREM2* missense mutations have been associated with congenital conditions such as Fraser syndrome [26] and unilateral renal agenesis [27]. According to the GeneCards database [25], low levels of FREM2 expression at the protein level have been detected in the Ga-MG cell line one of conventional GB cell lines [25].

#### 2.5.2. SPRY1—Expression Patterns and Association with Disease

SPRY1 is a protein with cellular location dependent on cell activation via epidermal growth factor (EGF). In cells not stimulated by EGF, it is localized mainly in the cytoplasm, while in stimulated cells it migrates to the edge of the outer cell membrane where it becomes anchored as a peripheral membrane protein. SPRY1 has been reported to inhibit the differentiation of murine NSC into neurons, so it most likely acts in the same way with regard to human embryonic NSC, where its expression has already been confirmed [28]. There are no reports of SPRY1 detection in NSC in the adult brain [29].

Beside its pathological role in non-malignant legius syndrome—a condition similar to neurofibromatosis—and alongside its involvement in glioma (which will be discussed later) SPRY1 has pathological role in non-malignant Legius syndrome and has been implicated in some malignancies, such as adrenal cortical adenocarcinoma, prostate cancer, and rhabdomyosarcoma (RMS) [29,30]. In RMS, SPRY1 is present in cancer stem cells [30].

#### 2.5.3. Reported Relations between Glioblastoma Multiforme and FREM2/SPRY1

FREM2 and SPRY1 were briefly mentioned in relation to GB in a few studies. However, none of these studies was focused on identification of new GB/GSC biomarkers. Ivliev et al. [31] identified EGFR gene amplification as a genetic signature in glioma and suggested its cause was the EGF signaling deregulation mediated by Sprouty family genes. Since *SPRY1* is a member of this family, it may be implicated alongside other related genes in glioma carcinogenesis. Likewise, *FREM2* gene amplification [32] has been discovered in gliosarcoma, a mixed tumor with a glioblastoma multiforme-resembling glial component and a sarcoma-like mesenchymal component. The mesenchymal part contains glial cells that have acquired mesenchymal properties by mutation. *FREM2* amplification is part of genetic signature of these cells and may be causatively linked to glial–mesenchymal transformation [32]. 

In a study by Forte et al. [33], *SPRY1* was among a number of genes with elevated expression in the undifferentiated glioblastoma-derived neuropsheres as compared to the differentiated glioblastoma-derived primary cultures. An unrelated study Aldaz et al. [34] demonstrated that SPRY1 was over-expressed at the protein level in undifferentiated cells as opposed to cells that had undergone four days of differentiation. Expression of SPRY1 protein was decreased in undifferentiated cells transfected with microRNA-21 (miR-21)—a small non-coding RNA that supposedly acts as a tumor suppressor—and the silencing of *SPRY1* mRNA by miR-21 was subsequently confirmed as the mechanism behind this decrease [34].

Berezovsky et al. [35] knocked down the expression of Sox2 and reported (among many other genes) decreased expression of *FREM2* in Sox2-negative glioblastoma-derived neurospheres, as well as decreased expression of *SPRY1* in Sox2-negative serum-differentiated glioblastoma cells. Recently, Okawa et al. [36] analyzed the proteomes and secreted proteomes (secretomes) of NSC and cells obtained from four GSCLs. FREM2 was found to be among 447 proteins differentially expressed between the proteomes of NSC and GSCL, as well as among 138 proteins differentially expressed between the corresponding secretomes. However, this study did not reveal which of the differentially expressed genes had elevated expression and which had decreased expression in GSCL. On the other hand, SPRY1 expression levels demonstrated no significant difference between the proteomes, and SPRY1 was not even detected in the secretomes. 

*SPRY2*, a gene from the Sprouty family—related but not identical to *SPRY1*—has already been proposed as a general biomarker of glioblastoma cells and a potential therapeutic target [37]. SPRY2 is a known regulator of oncogenic receptor tyrosine kinases (RTK), which are involved in signaling processes that regulate glioblastoma cell tumorigenicity and survival. Analysis of glioblastoma specimens [37] confirmed SPRY2 expression at the protein level in tumor tissue and further demonstrated that SPRY2 was over-expressed in glioblastoma tumors expressing EGFR variant III (EGFRvIII), a mutant EGFR variant that is constitutively active and whose presence in the tumor is deemed to be a negative prognostic indicator. As the Sprouty family genes are closely related, the function of *SPRY1* in glioblastoma carcinogenesis might resemble that of *SPRY2*. SPRY2 was also reported to be one of extracellular matrix components that prevent EGFR downregulation [38], and constitutive EGFR activation was in turn implicated as one of necessary steps for the transformation of non-malignant NSC into malignant GSC [39]. Non-malignant glial cells such as astrocytes can also give rise to GSC after EGFR activation [39]. Thus, elevated expression of extracellular matrix components such as Sprouty proteins and FREM2—whose amplification in glial cells has already been linked to their transformation into mesenchymal cells in gliosarcoma [32]—may be involved in the NSC–GSC and astrocytes–GSC transformations via prevention of EGFR deactivation.

### 2.6. Experimental Validation of the Identified Marker Candidates

#### 2.6.1. Quantitative Reverse Transcription Polymerase Chain Reaction (qRT-PCR)

Gene expression of *FREM2* and *SPRY1* was measured in the mature glioblastoma cell lines U251MG and U87MG, in the stem-like NCH cells—a co-culture of the glioblastoma stem-like cell lines NCH644 and NCH421K —as well as in neural stem cells (NSC) and astrocytes. mRNA was successfully isolated from all cell lines with mean concentration 1357 ng/µL and A_260_/A_280_ ratio from 1.82 to 1.99. Primer efficiency for *FREM2* was 1.71 and for *SPRY1* 1.87.

Statistical analysis of the results was performed in GraphPad Prism 6. All data sets were tested for normality (Gauss distribution) and outliners were identified. In the cases where no expression was observed (e.g., *FREM2* in U87MG and astrocytes) zeroes were added to the graph data sets. Outliers are presented on the graphs, but were removed using the ‘Identify outliers’ option of column analysis in GraphPad Prism 6 software and were thus excluded from the statistical analysis (one-way ANOVA, unpaired *t*-tests, Kruskal–Wallis, and Mann–Whitney tests).

**Figure 1:**
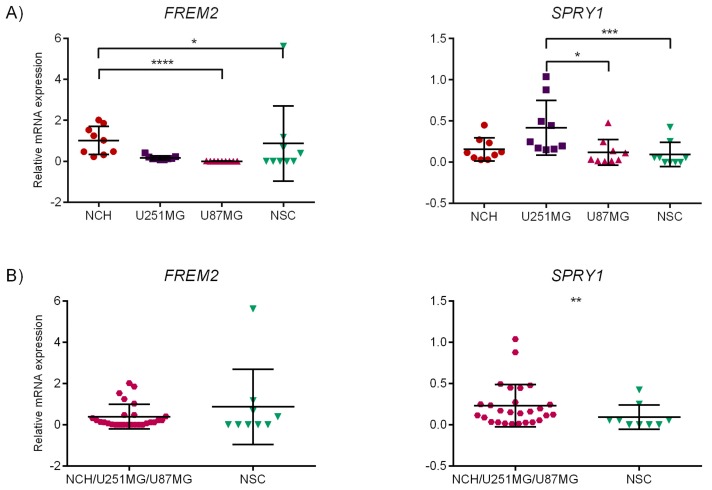

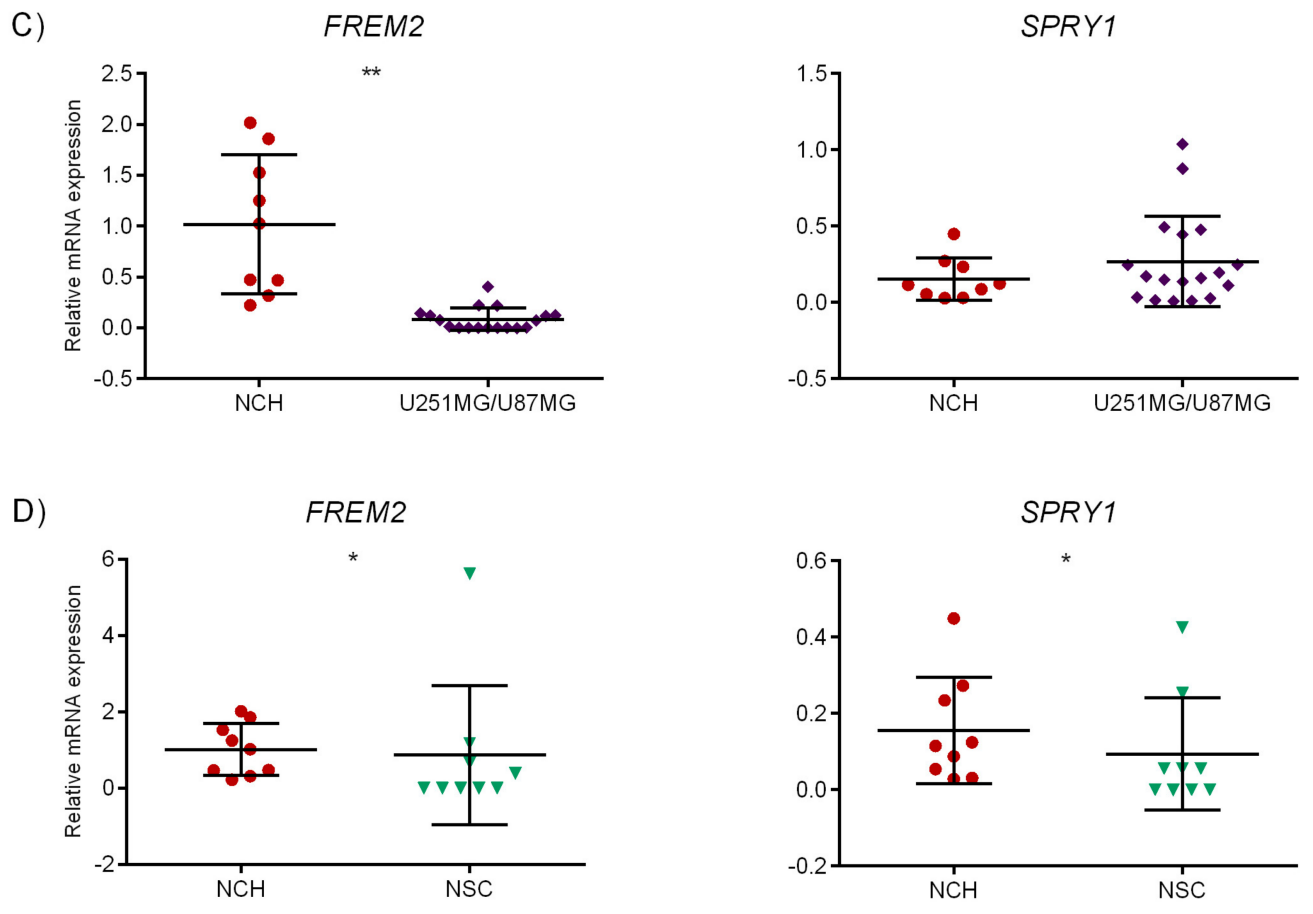
Statistical analysis of qRT-PCR results. Relative mRNA expression levels for *FREM2* and *SPRY1* for NCH, U251MG, U87MG, and NSC. Mean gene expression value is presented on the scatter plot (middle line) with error bars representing standard deviation (SD). *x*-axis: samples; *y*-axis: relative mRNA expression. * *p* ≤ 0.05, ** *p* < 0.01, *** *p* < 0.001, **** *p* < 0.0001. (**A**) Kruskal–Wallis test with Dunn’s correction: NCH vs. U87MG: *FREM2*—significant difference (*p* < 0.0001). NCH vs. NSC: *FREM2*—significant difference (*p* = 0.0489). U251MG vs. U87MG: *SPRY1*—significant difference (*p* = 0.0155). U251MG vs. NSC: *SPRY1*—significant difference (*p* = 0.0004). (**B**) Mann–Whitney test: NCH/U251MG/U87MG vs. NSC: *SPRY1*—significant difference (*p* = 0.0025). (**C**) Unpaired *t*-test with Welch’s correction: NCH vs. U251MG/U87MG: *FREM2*—significant difference (*p* = 0.0030). (**D**) Mann–Whitney test: NCH vs. NSC: *FREM2*—significant difference (*p* = 0.0181). NCH vs. NSC: *SPRY1*—significant difference (*p* = 0.0227).

Multiple group comparisons revealed a difference in *FREM2* expression between NCH and U87MG, as well as between NCH and NSC (Figure 1A, left). For *SPRY1*, significantly different expression was observed between U251MG and non-malignant neural stem cells (Figure 1A, right), as well as between both conventional glioblastoma cell lines, U251MG vs. U87MG (Figure 1A, right). Mann–Whitney test showed change in expression of *SPRY1* in NCH/U251MG/U87MG compared to NSC, whereas no change was observed for *FREM2* for the same analyzed groups (Figure 1B). Statistical analysis with unpaired *t*-test with Welch’s correction highlighted significant difference in *FREM2* expression between NCH and the mature glioblastoma cell lines U251MG and U87MG (Figure 1C, left). Comparison between NCH and NSC (Figure 1D) revealed that both *FREM2* and *SPRY1* are differentially expressed in malignant (NCH) when compared to non-malignant (NSC) stem cells.

**Figure 2:**
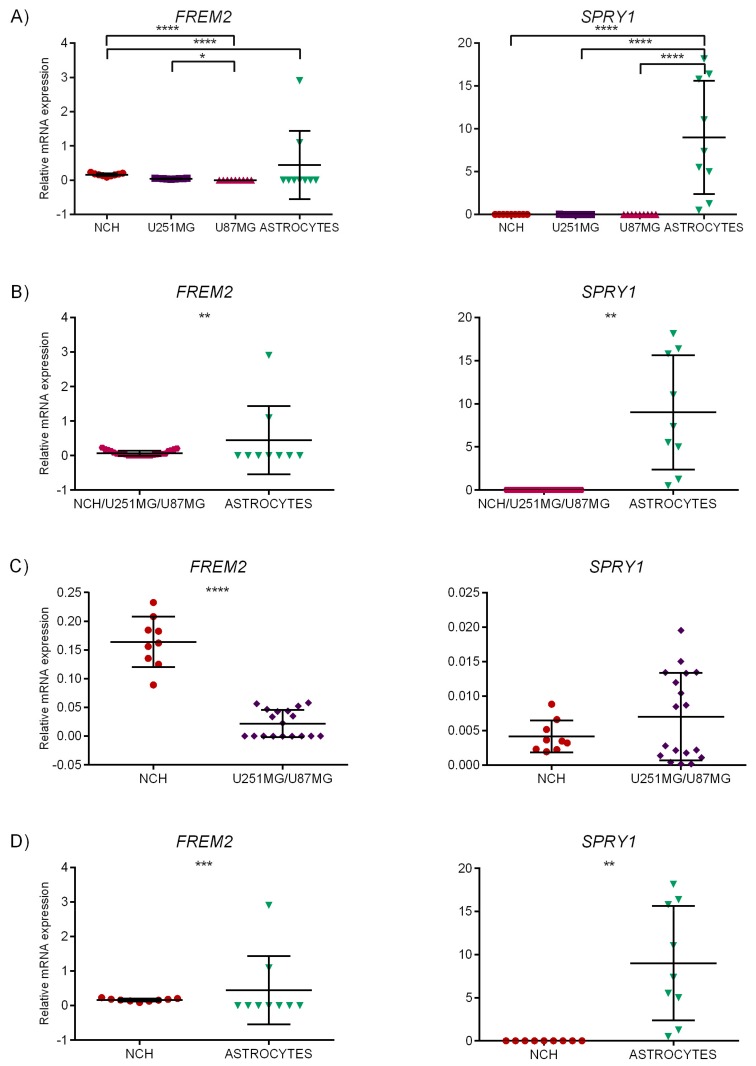
Statistical analysis of qRT-PCR results. Relative mRNA expression levels for *FREM2* and *SPRY1* for NCH, U251MG, U87MG, and astrocytes. Mean gene expression value is presented on the scatter plot (middle line) with error bars representing standard deviation (SD). *x*-axis: samples; *y*-axis: relative mRNA expression. * *p* ≤0.05, ** *p* < 0.01, *** *p* < 0.001, **** *p* < 0.0001. (**A**) Kruskal–Wallis test with Dunn’s correction: NCH vs. U87MG: *FREM2*—significant difference (*p* < 0.0001). NCH vs. astrocytes: *FREM2*—significant difference (*p* < 0.0001). U251MG vs. U87MG: *FREM2*—significant difference (*p* = 0.0295).One-way ANOVA multiple comparisons test: NCH vs. astrocytes: *SPRY1*—significant difference (*p* < 0.0001). U251MG vs. astrocytes: *SPRY1*—significant difference (*p* < 0.0001). U87MG vs. astrocytes: *SPRY1*—significant difference (*p* < 0.0001). (**B**) Unpaired *t*-test with Welch’s correction: NCH/U251MG/U87MG vs. astrocytes: *FREM2*—significant difference (*p* =0.0044).NCH/U251MG/U87MG vs. astrocytes: *SPRY1*—significant difference (*p* = 0.0036). (**C**) Mann–Whitney test: NCH vs. U251MG/U87MG: *FREM2*—significant difference (*p* < 0.0001). (**D**) Mann–Whitney test: NCH vs. astrocytes: *FREM2*—significant difference (*p* = 0.0002). Unpaired *t*-test with Welch’s correction: NCH vs. astrocytes: *SPRY1*—significant difference (*p* = 0.0036).

In a separate experiment, we analyzed all glioblastoma cell lines (NCH, U251MG, and U87MG) in combination with astrocytes as reference cells. Multiple group comparisons showed significant difference in expression of *FREM2* in NCH compared to astrocytes, between NCH and U87MG and between the two mature glioblastoma cell lines U251MG and U87MG (Figure 2A, left). For *SPRY1*, multiple group comparisons showed strong difference in expression of all glioblastoma cell lines (NCH, U251MG, and U87MG) compared to astrocytes where higher expression was observed (Figure 2A, right). For *FREM2*, Mann–Whitney test showed difference in expression of the combined glioblastoma cell lines (NCH, U251MG, and U87MG) compared to astrocytes, and unpaired *t*-test with Welch’s correction also showed change in expression of *SPRY1* for the tested groups. When comparing NCH to both U251MG and U87MG mature cell lines, significant change in expression was observed for *FREM2* only (Figure 2C). At last, comparison of NCH and astrocytes showed significant change in expression for both analyzed genes *FREM2* (overexpression in NCH) and *SPRY1* (overexpression in astrocytes) (Figure 2D).

These results indicate that *FREM2* was significantly over-expressed in glioblastoma stem-like cell line (NCH) in comparison to mature glioblastoma cells of the U87MG cell line representing relatively less aggressive neuronal type GB [40], and in comparison to neural stem cells and astrocytes. Its expression was also higher in the NCH cell line than in mature glioblastoma cells of the U251MG cell line representing most aggressive mesenchymal type GB [40]. In addition, *FREM2* was found significantly over-expressed in NCH in relation to the combination of mature GB-related cells of U87MG/U251MG, and significance of its over-expression was also found in U251MG compared to U87MG. Since the difference in *FREM2* expression between NCH and U251 was not significant, although *FREM2* expression was higher in the stem-like NCH cell line, this marker candidate may be considered a common indicator of aggressiveness of glioma cells.

*SPRY1* was expressed in all glioblastoma-related cell lines. *SPRY1* expression was significantly higher in the U251MG cell line towards both the U87MG and NSC cell lines (see Figure 1A, right). Its expression in glioblastoma-related malignant cells (NCH, U251MG, and U87 grouped together) was significantly higher than in the non-malignant NSC cell line (Figure 1B, right). However, it was also remarkably expressed in neural stem cells and especially in astrocytes. For both genes, relatively high expression levels were indicated in the U251MG cell line, which recapitulates the most typical pathobiological features reported for human GBM [40]. For *FREM2*, these experimental findings also confirm the results obtained by meta-analysis, namely the results of Test 5 (GSCL vs. conventional glioblastoma cell lines), where *FREM2* expression was significantly elevated in GSCL, with *p* < 0.001 and lfc > 1 (see Table 5).

#### 2.6.2. Immunofluorescent Staining

Gene transcription levels are not necessarily matched by the expression of corresponding proteins, which are the actual factors of physiological functions in the cell. Thus, with immunofluorescent detection, we validated the expression of FREM2 and SPRY1 proteins in the glioblastoma cell lines U251MG and U87MG compared to primary astrocytes as a control or a model of healthy environmental brain tissue.

**Figure 3:**
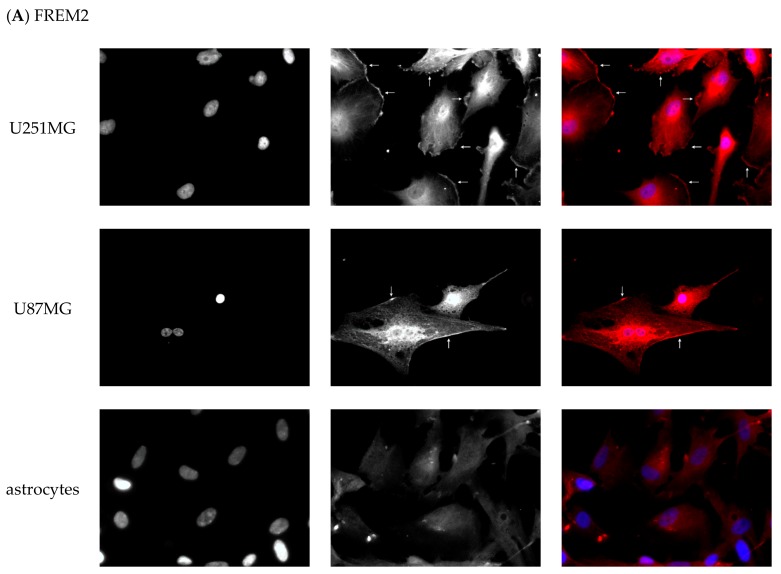

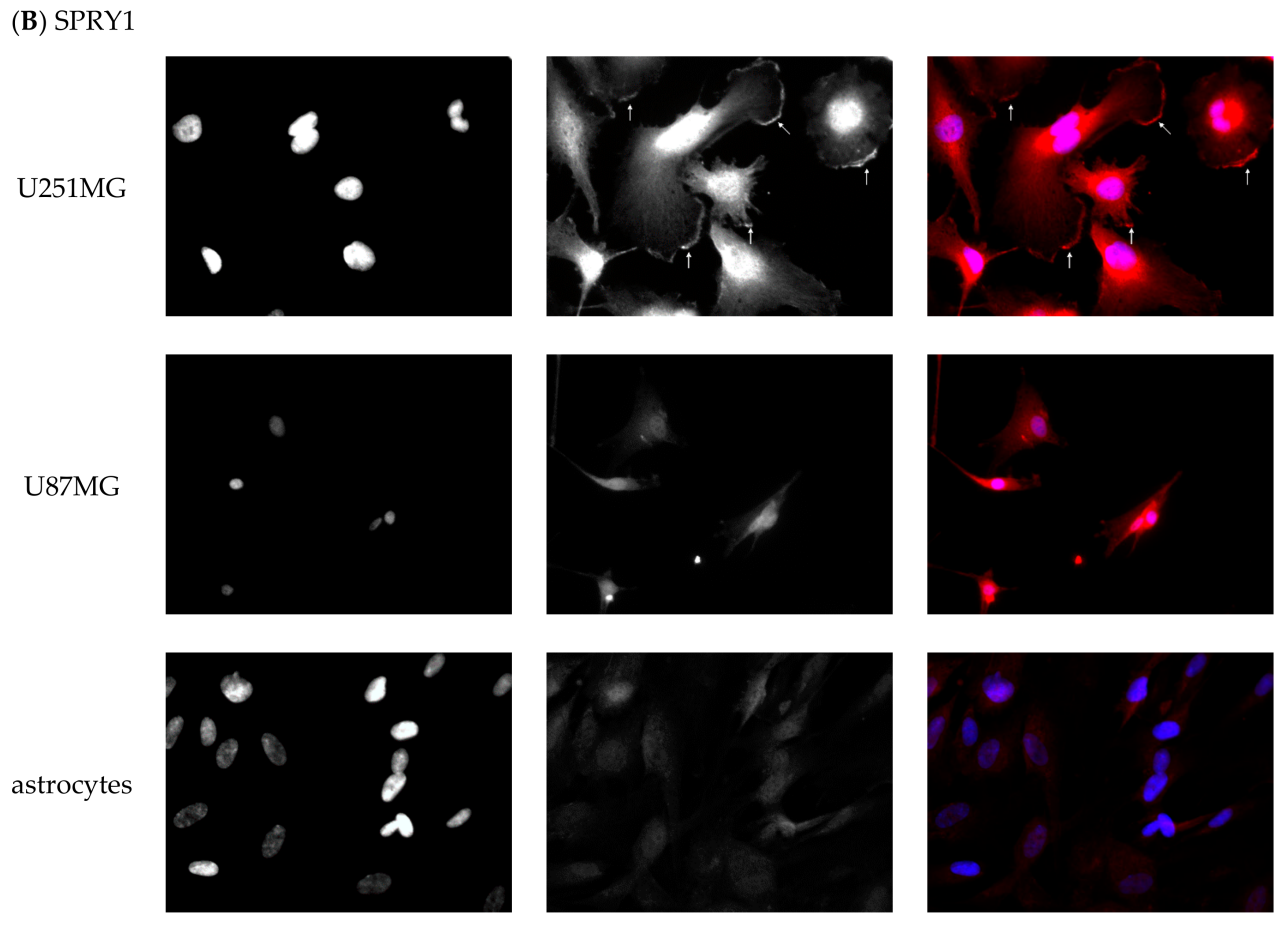
(**A**) Images of immunofluorescent staining of FREM2: nuclei (left); FREM2 staining with anti-FREM2 antibodies (middle); merged presentation with original staining (right): nuclei (blue); FREM2 (red). White arrows indicate staining of FREM2 in cell membranes; (**B**) Images of immunofluorescent staining of SPRY1: nuclei (left); SPRY1 staining with anti-SPRY1 antibodies (middle); merged presentation with original staining (right): nuclei (blue); SPRY1 (red). White arrows indicate staining of SPRY1 in cell membranes.

Experimental results are presented in Figure 3A,B. Cells were fixed in formaldehyde and immunofluorescently stained with commercially available anti–FREM2 (Figure 3A) and anti-SPRY1 (Figure 3B) antibodies. For better contrast, the left part of the image is shown in black and white, and the overlay in the original staining is shown on the right. Both target proteins are stained red, while the nuclei are blue. In the U251MG cell line, FREM2 was found highly expressed and clearly localized on the cell membrane, but also present close to the nuclear membrane (location of ER with ribosomes as the site of protein synthesis) and in cytosol, which may reflect significant overexpression of this protein in the aggressive U251MG cell line. Both cell membrane and cytosolic localization for FREM2 are in concordance with the data from the COMPARTMENTS database [25], while PROTEINATLAS (which also contains data from the U251MG cell line) [41] highlights its cytosolic and UNIPROT [42] its membrane localization. Differences in data from individual databases are probably due to different biological sources (cells, tissues). In our case, FREM2 was less membrane-expressed in the less aggressive U87MG cell line, and much less in non-malignant astrocytes, where no evidence of its membrane localization was found. SPRY1 was also found to be localized on the membrane of U251MG cells while no evidence of membrane localization was found in U87MG and non-malignant astrocytes.

These results highlighted FREM2 and potentially also SPRY1 as GBM marker candidates that may be useful for therapeutic targeting since they are highly expressed on the surface of the most typical GBM cells while not expressed on the surface of non-malignant glial cells which are found in tumor surroundings.

#### 2.6.3. Western Blot Validation

**Figure 4:**
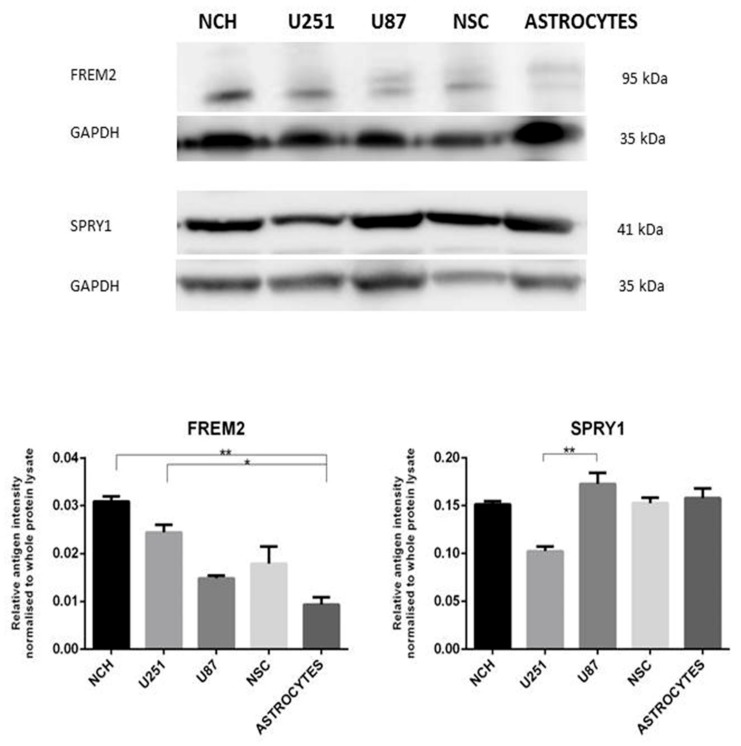
Western blot analysis. NCH—protein extract from glioblastoma stem-like cells, NCH cell line (NCH644 & NCH421K). U251, U87—protein extracts from (respectively) glioblastoma U251MG and U87MG cell lines. NSC—protein extract from neural stem cell cells. ASTROCYTES—protein extract from primary human astrocytes. Quantification of Western blotting for FREM2 and SPRY1 expression: relative band intensities were calculated as the ratios between the antigen band intensities for FREM2 and SPRY1 and those of the whole protein loaded on the gel (both measured in arbitrary units [AU]). GAPDH was used as loading control for both western blots. Statistical analysis of Western blot. Mean protein expression value is presented on the graph with error bars representing standard deviation (SD). *x*-axis: samples; *y*-axis: relative protein expression. * *p* ≤0.05, ** *p* < 0.01. Kruskal-Wallis test with Dunn’s correction: NCH vs. ASTROCYTES: FREM2—significant difference (*p* = 0.0013); U251 vs. ASTROCYTES: FREM2—significant difference (*p* = 0.0405); U251MG vs. U87MG: SPRY1—significant difference (*p* = 0.0027).

In order to support and quantify the results obtained with immunofluorescent staining and considering qRT-PCR findings, and to more precisely define if either of the markers is more specific to glioblastoma stem cells, we performed Western blot analysis on all cell lines used in this study. The results—which for FREM2 broadly support the previous results from qRT-PCR analysis and immunofluorescent staining—are presented in Figure 4. FREM2 was significantly overexpressed in the aggressive mesenchymal U251MG cell line in comparison with non-malignant astrocytes, while no such observation could be made for the less aggressive neural U87MG cell line. FREM2 expression was also higher in NCH when compared to both U251MG and U87MG, even when considering confidence intervals, but these differences were not statistically significant. The results for FREM2 expression in astrocytes confirm the results previously obtained with RT-qPCR and immunofluorescence, which both showed very low expression levels that correlate with the literature data that indicate the absence of this marker in the adult brain [29]. Conversely, the results for SPRY1 confirmed its substantial expression at the protein level in astrocytes, which was also demonstrated at the gene expression level with qRT-PCR.

At the protein level, both FREM2 and SPRY1 were expressed in NSC, with no significant difference to NCH, although in the case of FREM2, the expression in NCH was remarkably higher than in NSC. Expression of both potential marker candidates in non-malignant neural stem cells is not unexpected, since it is also a common trait of established cell-surface GSC marker candidates, such as CD133. Western blot analysis confirmed qPCR results that revealed the presence of FREM2 and SPRY1 in neural stem cells and in stem-like glioblastoma cells. In addition, while expression of SPRY1 was found to be relatively high in all glioblastoma-related cell types, FREM2 expression was remarkably higher in GSC compared to U251 and especially U87, and higher than in NSC.

Western blot results indicate that FREM2 is promising GBM marker candidate for typical GB tumors, represented by the U251MG cells, but is less suitable as a marker for atypical, less aggressive GB tumors, represented by the U87MG cells. FREM2 could also be a potential GSC marker candidate even though expression differences in the comparisons NCH vs. U251MG and NCH vs. U87MG fell short of being statistically significant. Thus, our experimental validation confirmed FREM2 as a novel glioblastoma marker candidate, and additionally a potential GSC marker candidate. On the other hand, Western blot analysis did not support the findings of immunocytochemistry for SPRY1, which however still remains as a marker candidate to distinguish GB-related cells (lower expression) from non-malignant astrocytes (remarkably higher expression) and neural stem cells (lower expression) at the qRT-PCR level.

## 3. Materials and Methods

### 3.1. Selection of Glioblastoma Datasets

In order to find appropriate datasets, we searched the data repositories GEO (NCBI, Bethesda MD, USA) and ArrayExpress (EMBL-EBI, Hinxton, UK), as well as the GLIOMASdb database (Beijing Neurosurgical Institute, Beijing, China).

We used the following queries: (i) “glioblastoma” for GEO; (ii) “glioblastoma, grade” for ArrayExpress; (iii) “glioblastoma, stem cells” for ArrayExpress; and (iv) “glioblastoma, neurospheres” for ArrayExpress (note that ArrayExpress contains more datasets, so more detailed queries were necessary than for GEO). We applied the following filters: (i) “homo sapiens”, “DataSets” for GEO; and (ii) “homo sapiens”, “RNA assay”, “array assay” for ArrayExpress. No queries were necessary for the GLIOMASdb repository, which contains only one dataset.

We devised seven pairwise comparisons relevant for selection of new GB/GSC marker candidates (Table 1). Each dataset had to contain groups of samples that would permit at least one of these comparisons. Additionally, each compared group had to contain at least 10 samples (therefore, we considered only datasets with at least 20 samples, as this is the minimal number that would theoretically permit comparison of two groups with 10 samples each). This cut-off sample size is derived from the findings of Stretch et al. [43] that the results of differential gene expression analysis based on less than 20 samples (in two equal-sized groups) lack prediction accuracy.

GEO and ArrayExpress datasets that fulfilled the requirements from the previous paragraph (subsequently referred to as appropriate datasets) were divided into two categories: (i) datasets with samples from gliomas of different grade, and (ii) datasets with samples of neurospheres and/or GSCL. Within each category, datasets were ranked by the number of comparisons relevant for GB/GSC marker selection (Table 1) that can be made by using data from a particular dataset. If two or more datasets enabled the same number of comparisons, priority was given to curated datasets (i.e., GEO datasets marked with “GDS”), for which NCBI assures that background processing and measurement are consistent across the dataset, that all value measurements are calculated in an equivalent manner, and that samples are biologically and statistically comparable [44]. The best datasets from each category were chosen to be used for the selection stage, while all other appropriate datasets were used for bioinformatical validation. The ranking of datasets obtained by using the above criteria is presented in Table 2. The single GLIOMASdb dataset was also chosen for use in the selection stage since it is curated, enables one of the relevant comparisons, and has a large size (325 samples). 

### 3.2. Selection of New Marker Candidates

#### 3.2.1. Differential Gene Expression Analysis with GEO2R

In each test used for selection or validation (see Table 3)—except in the Test 8, which was based on GliomasDB, where results had already been provided by the dataset authors [18]—we used the program GEO2R (NCBI, Bethesda MD, USA) to find differentially expressed genes. The program GEO2R [45]—based on the limma R package [20]—was used to perform a series of two-tailed *t*-tests (see Table 3), which used variance stabilization [46] and FDR-corrected *p* values [47]. Additional data about the tests—such as detailed lists of samples included in each of the compared groups for each test, and graphical presentations of distribution of sample values (boxplots)—are available in the Supplements.

We used the cut-off values of *p* < 0.001 for FDR-adjusted *p* values and lfc > 1 (indicating the gene expression ratio of at least 2:1 between the two compared groups) for log change. A gene had to fulfill both the p value and lfc requirements in order to be listed as differentially expressed in an individual test. If the analyzed dataset included data from alternative probes that hybridize to the same gene transcript, a gene was deemed to fulfill the requirements if at least one of the hybridizing probes scored appropriate lfc and *p* values.

The lists of differentially expressed genes in each individual test were compared by the Draw Venn Diagrams [48] application (University of Ghent, Belgium). Genes were checked for localization of proteins that they encode (see the next subsection) and only those genes that encode cell-surface proteins (CSP genes) were taken into further consideration. CSP genes differentially expressed in 8/8 tests immediately qualified for further consideration, while CSP genes that passed 7/8 tests qualified if they had both FDR-adjusted *p* < 0.05 and lfc > 0 in the single failed test.

#### 3.2.2. Analysis of Localization Data of Corresponding Proteins with COMPARTMENTS

The database COMPARTMENTS [49] was used to obtain quantified localization data for proteins encoded by the genes that had been highlighted by the selection procedure. This database provides confidence scores that a protein can be found at a particular location, e.g., in the plasma membrane. A score of 5 denotes the highest probability, while a score of 0 indicates a complete lack of evidence for this location [49]. For each protein that corresponds to one of highlighted candidate genes, we looked for its COMPARTMENTS score indicating the probability of its localization at the membrane. If this score was high—i.e., 5 or 4—the protein was deemed to be a cell-surface protein (CSP).

### 3.3. Assessment of the Effectiveness of Selection Tests

We were interested in the performance of established GSC markers candidates in our selection tests, since established marker candidates are expected to demonstrate significantly elevated expression in any relevant test. Six established CSP-encoding GSC marker candidates (*CD133*/*PROM1*, *CXCR4*, *MELK*, *PTCH1*, *CD9*, and *CD44*) were used as positive control to check the relevance of selection tests. Housekeeping genes known for their stable expression either across all cancer types (HPRT1 [23] and EMC7 [50]) or particularly in glioma (CYC10 [51], H3F3A [52,53], and RPLP0 [52]) were used as negative control. 

A class-based point system was used to reflect different levels of significance (measured by *p* values) and elevated expression (measured by lfc). Based on *p* values and lfc, genes were classified into four classes: (i) a gene had both FDR-adjusted *p* < 0.001 and lfc > 1; (ii) a gene did not meet the first criteria but still scored both FDR-adjusted *p* < 0.05 and lfc > 0; (iii) a gene did not meet the second criteria but still had lfc > 0 and *p* < 0.05; (iv) a gene failed all the three criteria—i.e., it had either *p* > 0.05, or lfc < 0, or both. The classes (i) and (ii) contain genes with statistically significant expression differences under conditions of multiple testing (FDR-correction of *p* values), while the classes (iii) and (iv) contain genes with non-significant differences after FDR-correction is applied.

The system was designed to give additional weight to classes (i) and (ii), which contain genes with statistically significant expression differences after FDR-correction. Genes from the class (i) received 4 points, those from the class (ii) received 3 points, those form the class (iii) received 1 point, while genes from the class (iv) received 0 points. Since some test results for a particular gene may be missing (in most cases because the gene is not featured on the microarray platform that was used to generate the data on which the test is based), we used average points per test (APPT) instead of sum of points as the performance measure for tested genes. When the APPT for an individual gene was calculated, tests with missing values for the gene were disregarded.

### 3.4. Validation Tests with the Data from Other Datasets

These tests were based on the seven relevant comparisons listed in the section Selection of glioblastoma datasets. Test numbers refer to numbers used in this listing: e.g., test v3-2 is the second (2) validation (v) test based on comparison 3. These tests used data from all appropriate datasets (see Selection of glioblastoma datasets) that were not chosen for the selection stage (Table 2). Classification of test results (points-based system), as well as use of established candidates and negative controls were the same as in the performance section of established marker candidates in selection tests.

### 3.5. Novelty Test of Genes Identified as Potential Markers

To assess the novelty of genes identified as potential markers, for each gene revealed by the selection procedure, we used the query (“name of the candidate, glioblastoma”) in the Google Scholar browser (Google, Mountain View CA, USA; accessed on 14 November 2017) and browsed the top 20 results. If this search revealed that a gene had already been linked to glioblastoma carcinogenesis, the gene was excluded from further consideration.

### 3.6. Experimental Validation

#### 3.6.1. Cell Cultures

All cell lines were grown in an incubator at 37 °C and 5% CO_2_. Human astrocytes (Ixcells Biotech, San Diego, CA, USA) [54] were expanded in Astrocyte Medium supplemented with penicillin/ streptomycin and astrocyte supplement (ScienCell, San Diego, CA, USA). Cell culture vessels (TPP, Trasadingen, Switzerland) were pre-treated with poly-L-lysine. The h9-derived human NSC (ThermoFisher, Waltham, MA, USA) [55] ) were grown in KnockOutTM DMEM/F-12 Basal Medium supplemented with GlutaMAX, antibiotic antimycotic, 1 mL StemPro neural supplement, 20 ng/mL EGF, 20 ng/mL bFGF (ThermoFisher) and 1 U/mL heparin (Sigma-Aldrich, Saint Louis, MO, USA). NCH cells (a co-culture of the glioblastoma stem-like cell lines NCH644 [56] and NCH421K [57]; both provided by CLS, Eppelheim, Germany) were expanded as spheroids in Neurobasal Medium supplemented with GlutaMAX, antibiotic/antimycotic, B-27 supplement, 20 ng/mL bFGF, 20 ng/mL EGF (ThermoFisher) and 1 U/mL heparin (Sigma-Aldrich). The conventional GB cell lines U251MG (CLS) [58] and U87MG (ATCC, Manassas, VA, USA) [59] were expanded in high-glucose (4.5 g/L d-glucose) Dulbecco’s modified Eagle’s medium (DMEM; ThermoFisher), supplemented with 10% fetal bovine serum, 2 mM l-glutamine, and antibiotic/antimycotic solution (ThermoFisher).

#### 3.6.2. Quantitative Reverse Transcription Polymerase Chain Reaction (qRT-PCR)

mRNA was extracted using TRI reagent (Sigma Aldrich) from the NCH glioblastoma stem-like cell line, conventional glioblastoma cell lines U251MG and U87MG, NSC, and astrocytes. Concentrations were measured using NanoDrop ND-1000 (NanoDrop Technologies, Wilmington, DE, USA) and purity was determined through the A_260_/A_280_ and A_260_/A_230_ ratios. Before transcription, 2 µg mRNA of each sample were treated with RNase-free DNase recombinant I (Roche, Basel, Switzerland) for 15 min at 30 °C and 10 min at 75 °C.

Transcription was performed using Transcriptor Universal cDNA Master (Roche) for 5 min at 25 °C, 10 min at 55 °C, and 5 min at 85 °C. qRT-PCR was performed with the Roche LightCycler 480 platform. Nine repetitions were performed for each cell line. There were two series of experiments: the first with NSC and the second with astrocytes as control. Total reaction volume was 5 µL/well and consisted of 0.75 µL cDNA, 2.5 µL 2x LightCycler 480 SYBR Green I Master (Roche), 0.3 µL of each 2.5 µM primer, and 1.15 µL distilled H2O. The following thermal cycling was used: 10 s at 95 °C (pre-incubation), 20 s at 60 °C and 20 s at 72 °C (cycling) for 45 cycles, 5 s at 95 °C and 1 min at 65 °C (melting curve), 95 °C continuous, and 30 s at 4 °C (cooling).

The following primer pairs were used: *RPL13A* (F: CCT GGA GGA GAA GAG GAA AGA GA, R: TTG AGG ACC TCT GTG TAT TTG TCA A), *CYC1* (F: GAG GTG GAG GTT CAA GAC GG, R: TAG CTC GCA CGA TGT AGC TG), *FREM2* (F: TGA GCC AAC TGT GTT TAT TC, R: GTA TAA CAG ACC ACC ATC AAC) and *SPRY1* (F: CTT TGC ATT AGG ATT TCA GAT G, R: GGA TCA CAA CTA ACG AAC TG), F—forward, R—reverse [51,60]. Eficiency of all primer pairs was determined using the standard curve method with pooled cDNA. Relative quantification was performed as described by Vandesompele et al. [61].

GraphPad Prism 6 (GraphPad Software Inc., La Jolla, CA, USA) was used for statistical analysis. For samples not following Gaussian distribution, relative mRNA expression (for FREM2 and SPRY1: “NCH vs. U251MG vs. U87MG vs. NSC”, and FREM2 “NCH vs. U251MG vs. U87MG vs. astrocytes”) was calculated with Kruskal–Wallis test and Dunn’s multiple comparisons test. For samples following Gaussian distribution, relative mRNA expression (for *SPRY1*: “NCH vs. U251MG vs. U87MG vs. astrocytes”) was calculated with ONE-Way ANOVA. Samples were analyzed as “NCH/U251MG/U87MG vs. astrocytes” (*FREM2*), “NCH/U251MG/U87MG vs. NSC” (*SPRY1*), “NCH vs. U251MG/U87MG” (*FREM2*), “NCH vs. NSC” (*SPRY1*) and “NCH vs. astrocytes” (*FREM2*) using Mann–Whitney test. For samples following Gaussian distribution (“NCH/U251MG/U87MG vs. astrocytes” (*SPRY1*), “NCH/U251MG/U87MG vs. NSC” (*FREM2*), “NCH vs. U251MG/U87MG” (*SPRY1*/*FREM2*), “NCH vs. astrocytes” (*SPRY1*) and “NCH vs. NSC” (*FREM2*) unpaired Student’s *t*-test was performed. In all cases *p* ≤ 0.05 was considered statistically significant (*: *p* ≤0.05, **: *p* < 0.01, ***: *p* < 0.001, ****: *p* < 0.0001).

The outliers were removed using the ‘Identify outliers’ option of column analysis in GraphPad Prism 6 software. The ROUT method, which was recommended by the program and can identify one or more outliers, was used. The ROUT method is based on a false discovery rate (FDR). The *Q* value, which is the maximum allowed FDR, was set to 1%, which means that there was a 1% chance of falsely discovering an outlier. The graphs as presented in Figure 1 and Figure 2 contain all data that were obtained, including the identified outliers. However, these values were excluded during the statistical analysis (one-way ANOVA, unpaired *t*-tests, Kruskal–Wallis, and Mann–Whitney tests).

#### 3.6.3. Immunofluorescent Staining

6 × 10^4^ U251MG cells (CLS) [58] were seeded on coverslips and incubated for 24 h at 37 °C and 5% CO_2_. 1 × 10^5^ U87MG cells (ATCC) [59] and 1 × 10^5^ astrocytes (Ixcells Biotech) [54] were seeded on poly-D-Lys (Sigma-Aldrich) pre-treated coverslips and incubated for 48 h at 37 °C and 5% CO_2_. Afterwards cells were washed three times and fixed in 4% formaldehyde for 15 min at room temperature. The fixative was washed with PBS three times for 10 min. Cells were permeabilized with 0.1% Triton X-100 for 15 min. Subsequently, cells were incubated for one hour in 1% BSA. After removal of blocking buffer, anti-FREM2 antibody (SAB3500517 produced in rabbit; Sigma-Aldrich) was added in dilution 1:30 and anti-SPRY1 (WH0010252M1 produced in mouse; Sigma-Aldrich) was added in dilution 1:25. The cells were incubated overnight at 4 °C. Afterwards cells were washed three times for 10 min with PBS and incubated with anti-rabbit secondary antibody (1:250 dilution; SAB4600399, Sigma-Aldrich) and anti-mouse antibody (1:250 dilution; SAB4600396, Sigma-Aldrich) for one hour at room temperature. Cells were washed three times for 10 min with PBS, stained with 300 nM DAPI, washed again three times for 10 min in PBS and then mounted on slides with ProLong Glass Antifade (ThermoFisher). Axio Imager M2 (Zeiss, Oberkochen, Germany) equipped with ZEN software was used to acquire images. Images were analyzed using ImageJ [62].

#### 3.6.4. Western Blot Analysis

From all cultured cell lines proteins were extracted using commercially available kit ProteoExtract^®^ Transmembrane Protein Extraction Kit (Merck, Darmstadt, Germany and protein concentration was determined with BCA assay. The expression of FREM2 and SPRY1 at the protein level in the cell lines U251MG, U87MG, NCH, NSC and primary human astrocytes was examined using Western blot analysis. Approximately 20 µg of protein lysate from each cell line was denaturated in 2% SDS, 60 mM dithiothreitol, 60 mM Tris-hydrochloric acid (Tris-HCl, pH 6.8), and 0.1% bromophenol blue, and loaded onto a NuPAGE 4% to 12% Tris-glycine gel. After SDS-PAGE proteins were transferred onto polyvinyldidene fluoride (PVDF) membranes. The membranes were stained using Ponceau S to show total protein amount loaded on the gel and quantified with the Multi Gauge 3.2 software (Fuji, Tokyo, Japan). The PVDF membranes were blocked for 1 h at room temperature in PBS containing 5% non-fat dried milk, and incubated overnight at 4 °C in 1% PBS-milk containing the primary antibody (rabbit polyclonal anti-FREM2, 1:500 dilution Novus Biologicals, Littleton, CO, USA; or mouse monoclonal anti-SPRY1, 1:500 dilution; Sigma-Aldrich). The membranes were washed in 0.1% PBS-Tween, incubated with the secondary antibody (anti-rabbit/mouse IgG HRP-conjugated antibody; 1:5000 dilution; Sigma Aldrich) for 1 h at 4 °C, and washed in 0.1% PBS-Tween. Membranes were treated with SuperSignal West Pico Chemiluminescent Substrate (ThermoFisher). Bands were visualized using a LAS-4000 CCD camera (Fuji) and analyzed with the Multi Gauge 3.2 software (Fuji).

Experiments of Western blot were performed in triplicates. The data were analyzed for statistical significance using one-way ANOVA followed by Dunn’s multiple comparison tests. *P* values < 0.05 were considered to indicate statistical significance. The means ± standard deviation were calculated for all variables.

## 4. Conclusions

Meta-analysis of genome-scale expression data from three unrelated public repositories identified four genes as potential new CSP gene markers of glioblastoma multiforme. After bioinformatic validation, two—*FREM2* and *SPRY1*—qualified for experimental validation at the transcriptional and protein levels. At the protein level of expression,, FREM2 was significantly over-expressed in the GBM-relevant cells in comparison to non-malignant brain cells, which was also confirmed at the gene level by qRT-PCR. In addition, FREM2 was over-expressed at the protein level in glioblastoma stem-like cells in comparison to mature GBM cells as well as neural stem cells. Moreover, qRT-PCR results proved significantly elevated expression of *FREM2* in glioblastoma stem-like cells when compared to mature GBM cells. These results confirmed FREM2 as a novel GBM marker candidate and also as a potential GSC marker candidate. On the other hand, SPRY1 was not confirmed as a GSC marker candidate at the protein level. However, its strong expression in astrocytes in relation to the studied GB-related cell lines highlights its potential value to distinguish GB-related cells from the non-malignant tissue. 

Meta-analysis may have some disadvantages, such as heterogeneity of input data, different ways of data normalization, and the use of groups with a low number of samples. These shortcomings were avoided by using curated data that were uniformly normalized and by not using very small groups in our statistical comparisons. The results of meta-analysis have been experimentally validated with three different in vitro methods, which also have their own limitations, such as the heterogeneity of cell lines used for in vitro experiments. In our study, there was a disproportion between the results of gene/protein expression in both conventional GBM cell lines. However, in the U251MG cell line, which recapitulates the most typical pathobiological features reported for human GBM, the experimental results for FREM2 were consistent with the findings of bioinformatic analysis. It is also difficult to compare gene/protein expression in cell lines with different growth speed, such as GSCL (e.g., NCH) and NSC. Unlike the GSCL, NSC cells grow very slowly and large number of passages is needed in order to acquire enough material for comparative expression studies. The NSC population likely became heterogeneous due to slow growth and consisted of unevenly differentiated cells, and this prevented determination of statistically significant differences in FREM2 expression at the protein level.

In conclusion, we identified two new cell-surface glioblastoma marker candidates, FREM2 and SPRY1. At the protein level, which matters the most for potential therapeutic use of markers, FREM2 was significantly overexpressed in the GBM-relevant cells in comparison to non-malignant brain cells and is thus proposed GBM marker candidate. In addition, FREM2 was overexpressed in glioblastoma stem-like cells in comparison to mature GBM cells as well as neural stem cells which highlights this protein as a putative biomarker of glioblastoma stem cells. As concerning SPRY1, despite Western blot results, it may also be interesting as a therapeutic target since immunofluorescent staining, as in the case of FREM2, indicated its presence on the membranes of GB cells belonging to the aggressive mesenchymal type, while it was not present on the surface of non-malignant astrocytes, where it was expressed exclusively in the cytosol and close to the nuclear membrane. Thus, SPRY1 could also be used as a target for therapeutic antibodies, which act by binding with cell-surface antigens and do not penetrate in cell interior. In order to assess the potential value of FREM2 and SPRY1 as GB/GSC-specific therapeutic targets, further research is needed at the level of immunohistochemistry and animal models. In this context, combination of both markers may be considered in research towards a multitargeting anti-glioblastoma therapeutic strategy.

## Figures and Tables

**Table 1:** Summary of dataset selection

Database	GEO	ArrayExpress			GLIOMASdb
Query	»glioblastoma«	»glioblastoma, grade«	»glioblastoma, stem cells«	»glioblastoma, neurospheres«	/
Filters	»homo sapiens«, »DataSets«	»homo sapiens«, »RNA assay«, »array assay«			/
All datasets	36	76	69	14	1
Datasets > 20 samples	11	58	17	3	1
Rejected—Reason 1	5	35	11	1	0
Rejected—Reason 2	2	16	3	2	0
Rejected—Reason 3	0	4	1	0	0
Appropriate datasets	4	3	1 + 1 conditionally *	0	1
Appropriate datasets (list)	GSE4412/GDS1975 GSE4412/GDS1976 GSE4290/GDS1962 GSE23806/GDS3885	E-GEOD-16011 E-GEOD-52009 E-GEOD-68848	E-GEOD-4536 E-GEOD-74571 *	/	GLIOMASdb

Reason 1—no relevant comparison possible (Comparisons relevant for selection of new GB/GSC marker candidates are: (1) glioblastoma multiforme tissue vs. non-malignant tumor tissue, (2) glioblastoma multiforme tissue vs. tissue from low-grade—i.e., grade II—gliomas, (3) glioblastoma multiforme tissue vs. tissue from oligodendrogliomas, (4) GSCL vs. ordinary glioblastoma multiforme tissue, (5) GSCL vs. CGCL, (6) neurospheres vs. ordinary glioblastoma multiforme tissue, (7) neurospheres vs. CGCL; the group in which elevated expression of GB/GSC markers is expected is marked in bold). Reason 2—relevant comparisons possible, but not with at least 10 samples in both compared groups. Reason 3—dataset is not original (duplicates or combinations of other GEO and ArrayExpress datasets). * two relevant comparisons possible: the first compares two groups with 9 samples each, the second compares a group of 10 samples with a group of 9 samples. Conditionally acceptable because this is the only found dataset that enables comparisons including glioblastoma neurospheres.

**Table 2:** Ranking of appropriate datasets

Dataset	Category	NPC	Rank	Curated	Use of Dataset
GSE4412/GDS1975	Grade	1	5	yes	validation
GSE4412/GDS1976	Grade	1	5	yes	validation
GSE4290/GDS1962	Grade	3	1 ***	yes	selection (winner)
E-GEOD-16011	Grade	2	3	no	validation
E-GEOD-52009	Grade	2	3	no	validation
E-GEOD-68848	Grade	3	1 ***	no	validation
GSE23806/GDS3885	Neuro/GSCL	4	1	yes	selection (winner)
E-GEOD-4536	Neuro/GSCL	2	2	no	validation
E-GEOD-74571	Neuro/GSCL	2	2	no	validation
GLIOMASdb	Grade	1	/	yes	selection (directly qualified)

Category: Grade—datasets with samples from gliomas of different grade; Neuro/GSCL—datasets with samples of neurospheres and/or GSCL; NPC = number of possible comparisons; Rank—NPC-based rank within category; *** If rank is equal, curated datasets have precedence.

**Table 3:** Summary of statistical tests

Stage	Test	First Group (Overexpression of GSC Marker Candidates Expected)		Second Group		DATA ORIGIN	
		Tissue/Cells	N	Tissue/Cells	N	Database	Dataset
Selection	1	GB	77	Non-malignant brain tissue	23	GEO	GSE4290/GDS1962
	2	GB	77	Grade II glioma	45	GEO	GSE4290/GDS1962
	3	GB	77	oligodendroglioma	50	GEO	GSE4290/GDS1962
	4	GSCL	27	GB tumor tissue	12	GEO	GSE23806/GDS3885
	5	GSCL	27	Conventional GB cell lines	32	GEO	GSE23806/GDS3885
	6	neurospheres	17	GB tumor tissue	12	GEO	GSE23806/GDS3885
	7	neurospheres	17	Conventional GB cell lines	32	GEO	GSE23806/GDS3885
	8	GB	143	Grade II glioma	110	GLIOMASdb	/
Validation	v1-1	GB	228	Non-malignant brain tissue	28	ArrayExpress	E-GEOD-68848
	v2-1	GB	24	Grade II glioma	63	ArrayExpress	E-GEOD-52009
	v2-2	GB	134	Grade II glioma	99	ArrayExpress	E-GEOD-68848
	v2-3	GB	159	Grade II glioma	24	ArrayExpress	E-GEOD-16011
	v3-1	GB	59	oligodendroglioma	11	GEO	GSE4412/GDS1975
	v3-2	GB	59	oligodendroglioma	11	GEO	GSE4412/GDS1976
	v3-3	GB	24	oligodendroglioma	13	ArrayExpress	E-GEOD-52009
	v3-4	GB	228	oligodendroglioma	67	ArrayExpress	E-GEOD-68848
	v3-5	GB	159	oligodendroglioma	52	ArrayExpress	E-GEOD-16011
	v4-1	GSCL	28	GB tumor tissue	17	ArrayExpress	E-GEOD-4536
	v5-1	GSCL	28	Conventional GB cell lines	26	ArrayExpress	E-GEOD-4536
	v6-1	neurospheres	9	GB tumor tissue	9	ArrayExpress	E-GEOD-74571
	v7-1	neurospheres	9	Conventional GB cell lines	10	ArrayExpress	E-GEOD-74571

N = number of samples, GB = glioblastoma.

**Table 4:** Selection of new GB/GSC marker candidates—top 10 genes.

Gene	Success Rate	CSP Status	Already Linked to Glioblastoma Carcinogenesis	Single Failed Test (*p* > 0.001 or lfc < 1)	Results of the Failed Test (Only for CSP Genes with 7/8 Success Rate)	
					*p* < 0.05	lfc > 0
***CD276***	**8/8**	**yes**	**yes** [19]	**none**	/	/
*HIST1H2BH*	8/8	no	no	none	/	/
*DUSP6*	8/8	no	yes [20]	none	/	/
*YME1L1*	7/8	no	yes [21]	Test 8	/	/
*DHRSX*	7/8	no	no	Test 8	/	/
*B3GNT5*	7/8	no	no	Test 7	/	/
***FREM2***	**7/8**	**yes**	**no**	**Test 6**	**yes**	**yes**
*DUSP4*	7/8	no	yes [22]	Test 3	/	/
***SPRY1***	**7/8**	**yes**	**no**	**Test 3**	**yes**	**yes**
***SLC47A1***	**7/8**	**yes**	**no**	**Test 1**	**yes**	**yes**

Genes selected for further analysis are shown in bold.

**Table 5:** Comparison of new GB/GSC marker candidates with established GSC marker candidates

Test	Points														
	New Candidates				Established Candidates						Control				
	*CD 276*	*FREM2*	*SPRY1*	*SLC47A1*	*CD133*	*CXCR4*	*MELK*	*PTCH1*	*CD9*	*CD44*	*CYC1*	*HPRT1*	*H3F3A*	*RPLP0*	*EMC7*
1	4	4	4	3	3	4	4	3	3	4	0	0	4	4	0
2	4	4	4	4	3	4	4	0	3	4	0	0	0	0	0
3	4	4	3	4	3	4	4	0	3	4	0	0	0	0	0
4	4	4	4	4	0	0	4	3	0	3	0	4	0	0	0
5	4	4	4	4	3	4	0	3	3	0	0	0	3	0	0
6	4	3	4	4	0	0	3	3	0	1	0	3	0	0	3
7	4	4	4	4	3	4	0	3	3	0	0	0	0	3	0
8	4	4	4	4	/	4	4	0	/	4	/	/	0	0	0
APPT	4.000	3.875	3.875	3.875	2.143	3.000	2.875	1.875	2.143	2.500	0.000	1.000	0.875	0.875	0.375

APPT = average points per test (rounded to three decimals); 4 points: FDR-corrected *p* < 0.001 and lfc > 1 (highly significant and highly overexpressed); 3 points: FDR-corrected *p* < 0.05 and lfc > 0, while criteria for 4 points are not met (significant and overexpressed); 1 point: *p* < 0.05 and lfc > 0 (overexpressed but borderline non-significant after application of FDR correction); 0 points: either *p* > 0.05 or lfc < 0 (non-significant or not overexpressed); /: gene not featured on the platform or in the results table.

**Table 6:** Results of bioinformatic validation tests

Test	Points														
	New Candidates				Established Candidates						Control				
	*CD 276*	*FREM2*	*SPRY1*	*SLC47A1*	*CD133*	*CXCR4*	*MELK*	*PTCH1*	*CD9*	*CD44*	*CYC1*	*HPRT1*	*H3F3A*	*RPLP0*	*EMC7*
v1-1	4	4	4	4	3	4	4	0	3	4	0	0	3	4	0
v2-1	3	/	3	3	0	0	4	0	0	3	0	0	3	0	0
v2-2	3	4	4	4	3	3	4	0	3	4	0	3	0	0	0
v2-3	3	/	4	4	3	4	4	0	3	4	0	3	/	/	/
v3-1	/	/	0	0	0	1	1	0	0	1	0	0	0	0	0
v3-2	3	0	3	/	/	/	/	0	0	0	/	/	/	/	0
v3-3	3	/	0	3	0	0	3	1	0	3	0	0	1	0	0
v3-4	3	3	3	3	3	3	4	0	3	4	0	3	0	0	0
v3-5	3	/	3	0	4	4	3	0	3	4	0	0	/	/	/
v4-1	4	4	4	0	0	0	4	0	0	0	3	0	0	3	3
v5-1	3	4	4	4	4	4	0	4	3	0	0	0	0	0	0
v6-1	3	3	1	3	0	0	0	0	3	0	3	0	0	3	3
v7-1	3	4	4	4	3	4	0	3	1	0	3	0	3	1	0
APPT	3.167	3.250	2.846	2.667	1.917	2.250	2.583	0.615	1.692	2.077	0.750	0.750	1.000	1.100	0.545

APPT = average points per test (rounded to three decimals). Legend from Table 4 also applies here.

## Supplementary Materials

Supplementary materials can be found at http://www.mdpi.com/1422-0067/19/5/1369/s1, Table S1: Results of Test 1; Table S2: Results of Test 2; Table S3: Results of Test 3; Table S4: Results of Test 4; Table S5: Results of Test 5; Table S6: Results of Test 6; Table S7: Results of Test 7; Table S8: Results of Test 8; Table S9: Results of Test v1-1; Table S10: Results of Test v2-1; Table S11: Results of Test v2-2; Table S12: Results of Test v2-3; Table S13: Results of Test v3-1; Table S14: Results of Test v3-2; Table S15: Results of Test v3-3; Table S16: Results of Test v3-4; Table S17: Results of Test v3-5; Table S18: Results of Test v4-1; Table S19: Results of Test v5-1; Table S20: Results of Test v6-1; Table S21: Results of Test v7-1; Table S22: Platform decoding for tests v2-3 and v3-5; Table S23: Results of Venn diagrams analysis; Figure S1: Selection of samples and boxplot for Test 1; Figure S2: Selection of samples and boxplot for Test 2; Figure S3: Selection of samples and boxplot for Test 3; Figure S4: Selection of samples and boxplot for Test 4; Figure S5: Selection of samples and boxplot for Test 5; Figure S6: Selection of samples and boxplot for Test 6; Figure S7: Selection of samples and boxplot for Test 7; Figure S8: Selection of samples and boxplot for Test 8; Figure S8: Selection of samples and boxplot for Test v1-1; Figure S9: Selection of samples and boxplot for Test v2-1; Figure S10: Selection of samples and boxplot for Test v2-2; Figure S11: Selection of samples and boxplot for Test v2-3; Figure S12: Selection of samples and boxplot for Test v3-1; Figure S13: Selection of samples and boxplot for Test v3-2; Figure S14: Selection of samples and boxplot for Test v3-3; Figure S15: Selection of samples and boxplot for Test v3-4; Figure S16: Selection of samples and boxplot for Test v3-5; Figure S17: Selection of samples and boxplot for Test v4-1; Figure S18: Selection of samples and boxplot for Test v5-1; Figure S19: Selection of samples and boxplot for Test v6-1; Figure S20: Selection of samples and boxplot for Test v7-1.

## Abbreviations

ANOVA	analysis of variance
APPT	average points per test
BSA	bovine serum albumin
CGCL	conventional glioblastoma cell lines
CSP	cell-surface protein
CSP gene	gene that encodes a cell-surface protein
CYC1	cytochrome c1
EGF(R)	epidermal growth factor (receptor)
EMBL-EBI	European Bioinformatics Institute
EMC7	endoplasmatic reticulum membrane protein complex subunit 7
FDR	false discovery rate
GB	glioblastoma multiforme
GEO	Gene Expression Omnibus
GPCR	G-protein coupled receptor
GSC	glioblastoma stem cells
GSCL	glioblastoma stem-like cell lines
H3F3A	H3 histone family 3A
HPRT1	hypoxanthine phosphoribosyltransferase 1
lfc	log2 of the ratio of average expression levels
limma	Linear Models for Microarray Analysis
MAPK	mitogen-activated protein kinase
miR	micro ribonucleic acid (micro RNA)
NCBI	National Center for Biotechnology Information
NSC	neural stem cells (non-malignant)
PBS	phosphate buffer saline
PI3K	phosphatidylinositol-4,5-bisphosphate 3-kinase
PTEN	phosphatase and tensin homolog
qRT-PCR	quantitative real-time polymerase chain reaction
RMS	Rhabdomyosarcoma
RPLP0	ribosomal protein lateral stalk subnit P0
RTK	receptor tyrosine kinase
