# Supplementary figures and images for: Meta-Analysis and Experimental Validation Identified FREM2 and SPRY1 as New Glioblastoma Marker Candidates
Source: Int J Mol Sci. 2018 May 4;19(5):1369. doi: 10.3390/ijms19051369 (PMC5983642; doi:10.3390/ijms19051369)

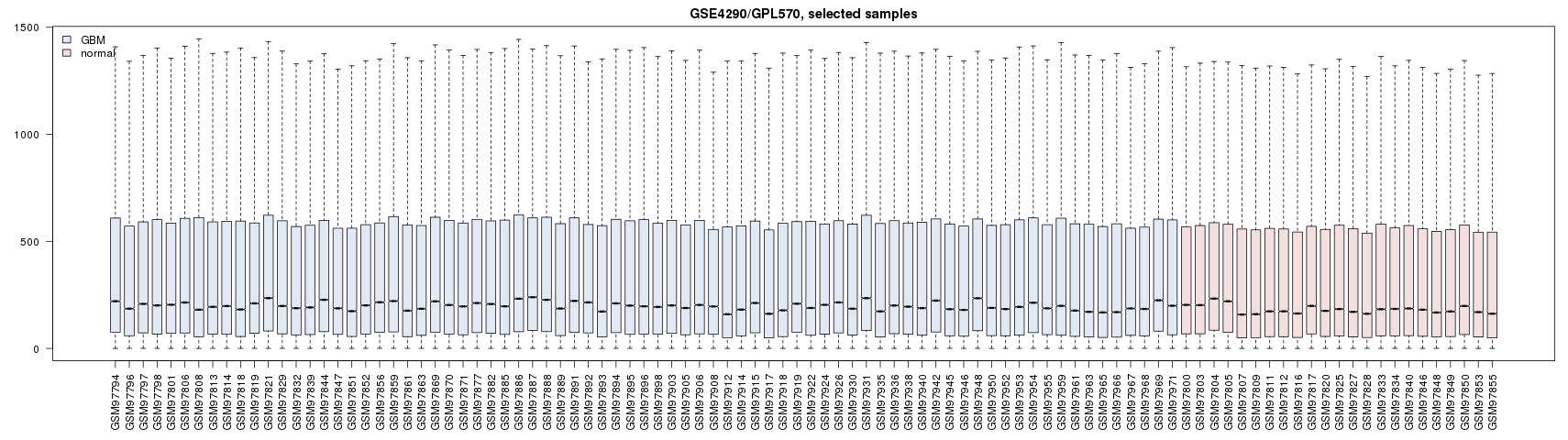

Supplement: Supplementary file 1 [file ijms-19-01369-s001.zip › Supplementary_files/Figure S1 test 1.png]

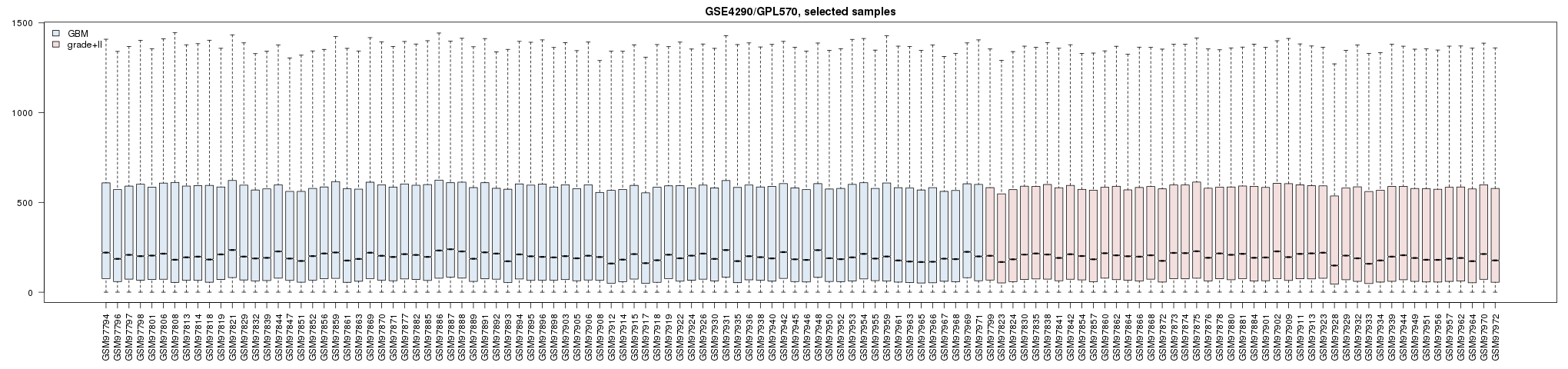

Supplement: Supplementary file 1 [file ijms-19-01369-s001.zip › Supplementary_files/Figure S2 test 2.png]

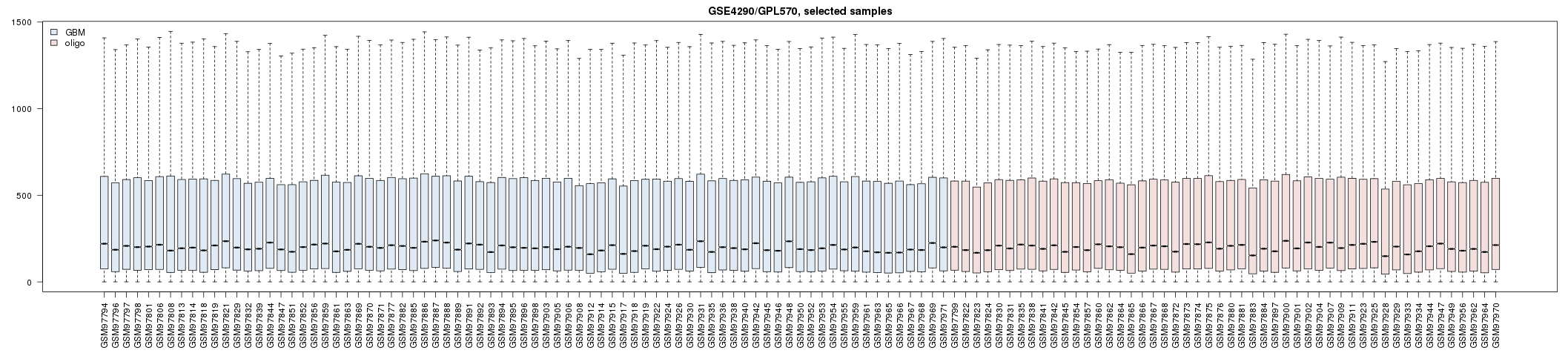

Supplement: Supplementary file 1 [file ijms-19-01369-s001.zip › Supplementary_files/Figure S3 test 3.png]

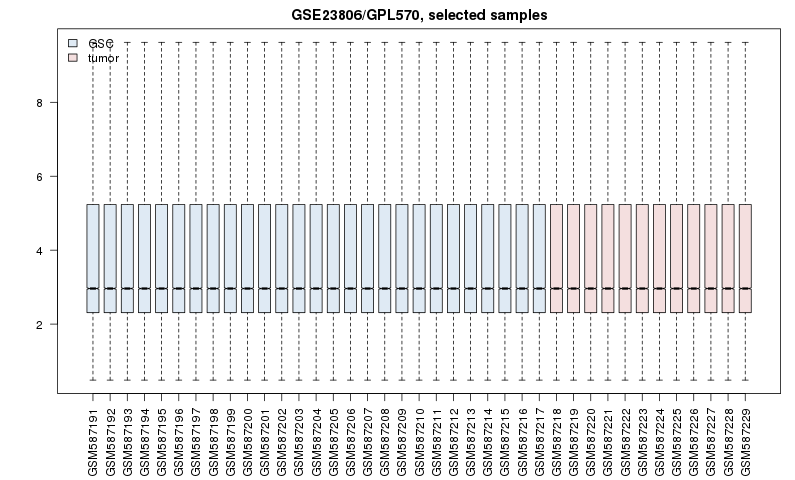

Supplement: Supplementary file 1 [file ijms-19-01369-s001.zip › Supplementary_files/Figure S4 test 4.png]

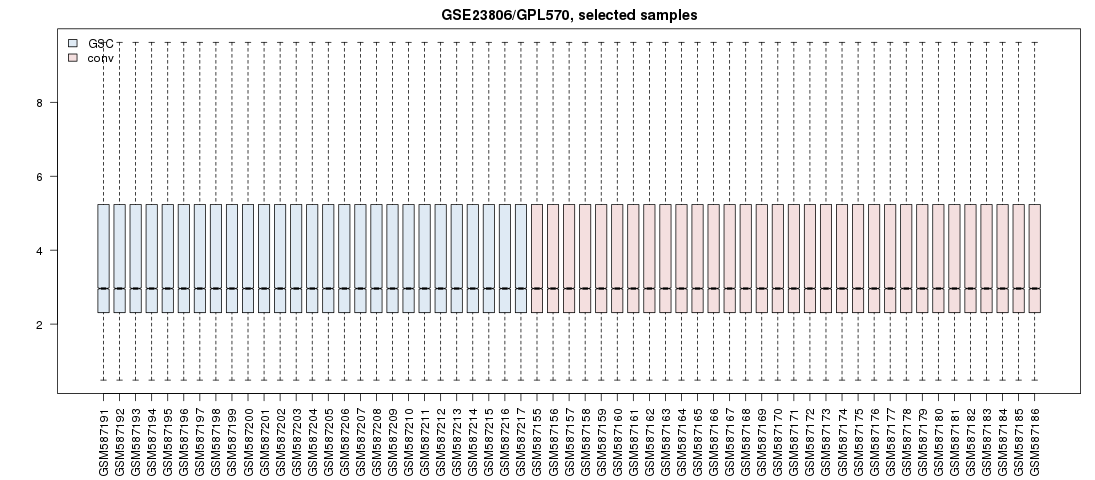

Supplement: Supplementary file 1 [file ijms-19-01369-s001.zip › Supplementary_files/Figure S5 test 5.png]

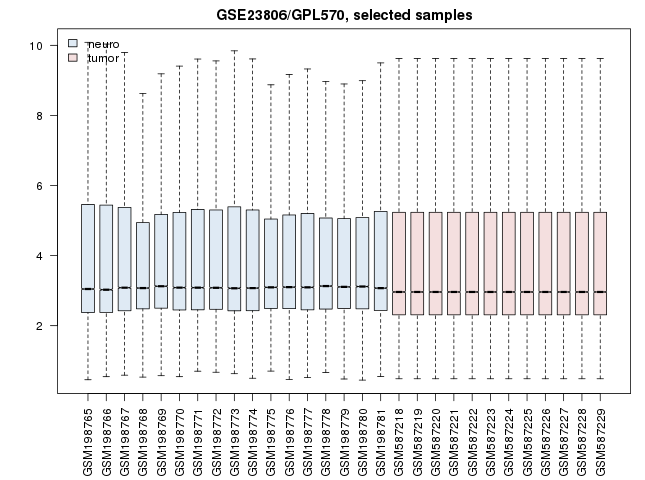

Supplement: Supplementary file 1 [file ijms-19-01369-s001.zip › Supplementary_files/Figure S6 test 6.png]

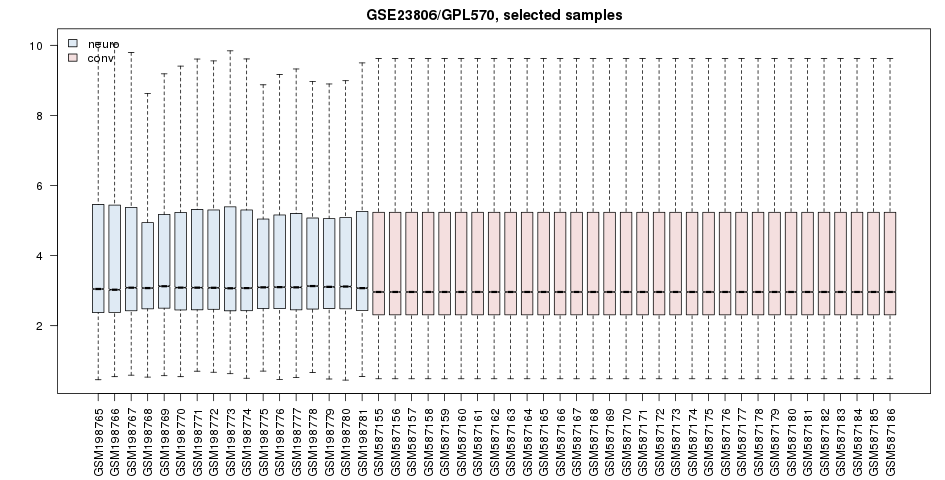

Supplement: Supplementary file 1 [file ijms-19-01369-s001.zip › Supplementary_files/Figure S7 test 7.png]

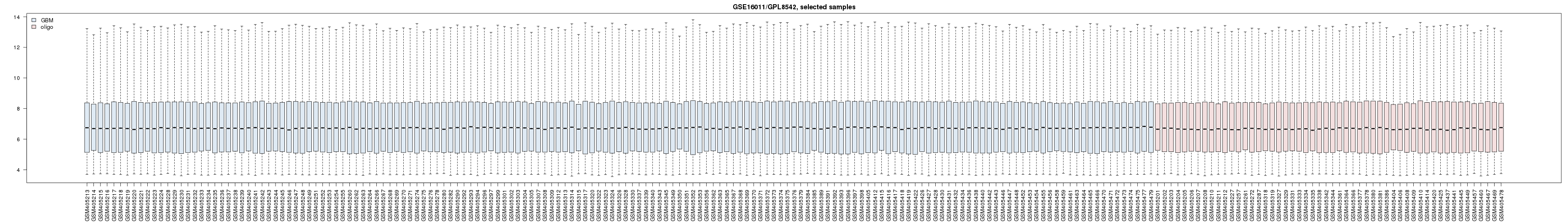

Supplement: Supplementary file 1 [file ijms-19-01369-s001.zip › Supplementary_files/Figure S16 test v3-5.png]

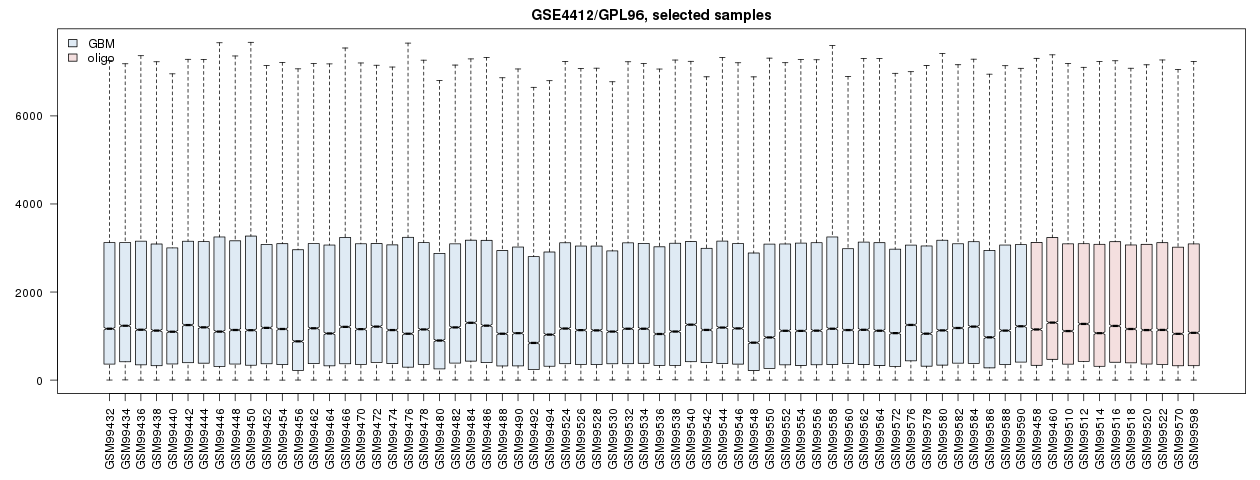

Supplement: Supplementary file 1 [file ijms-19-01369-s001.zip › Supplementary_files/Figure S12 test v3-1.png]

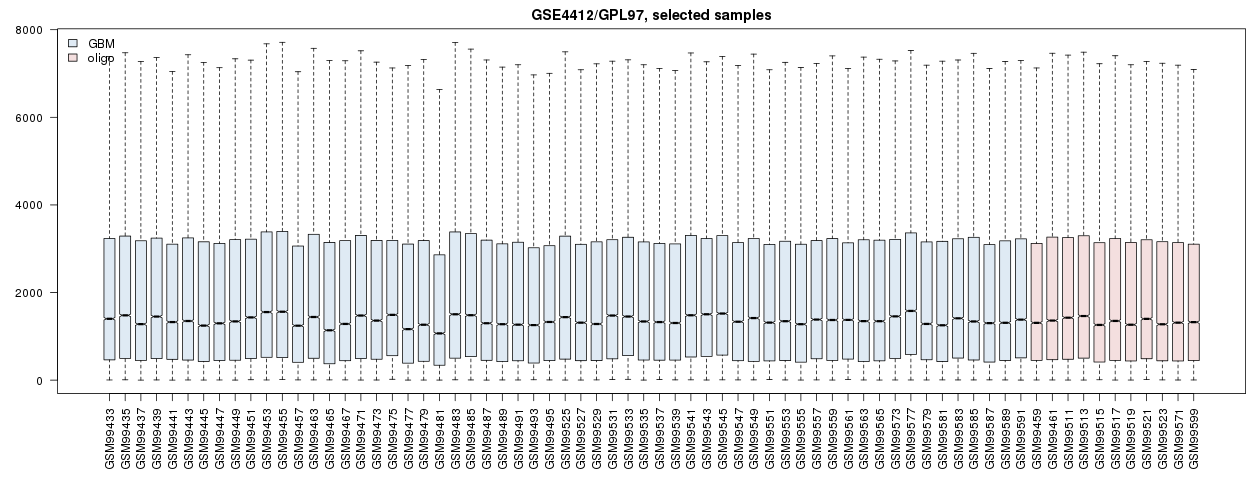

Supplement: Supplementary file 1 [file ijms-19-01369-s001.zip › Supplementary_files/Figure S13 test v3-2.png]

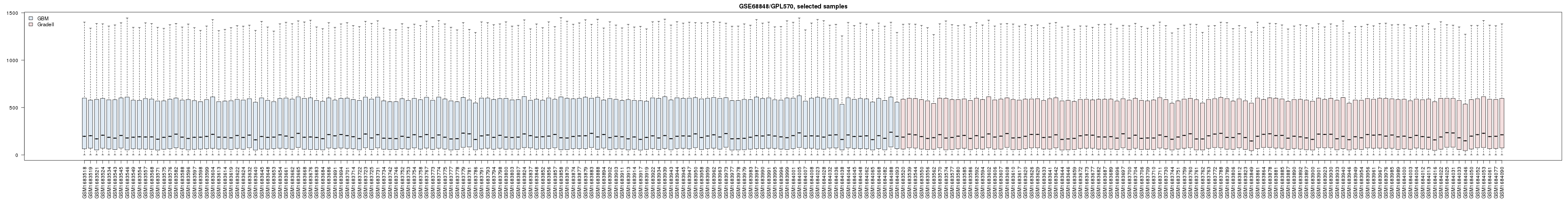

Supplement: Supplementary file 1 [file ijms-19-01369-s001.zip › Supplementary_files/Figure S10 test v2-2.png]

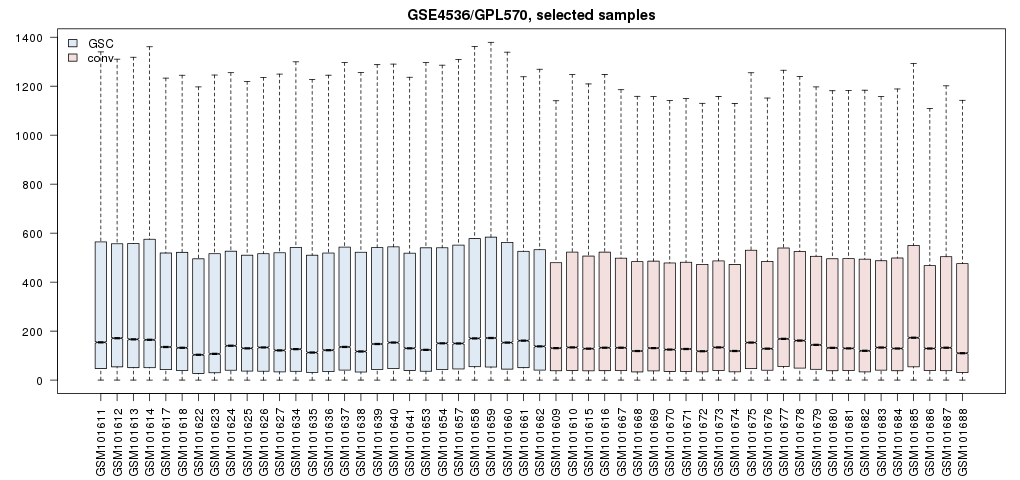

Supplement: Supplementary file 1 [file ijms-19-01369-s001.zip › Supplementary_files/Figure S18 test v5-1.png]

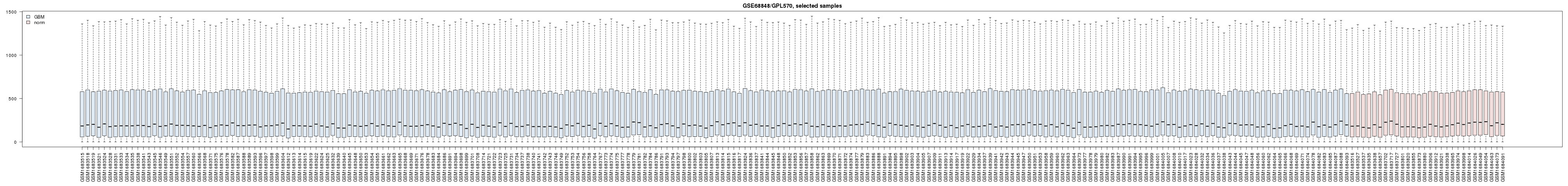

Supplement: Supplementary file 1 [file ijms-19-01369-s001.zip › Supplementary_files/Figure S8 test v1-1.png]

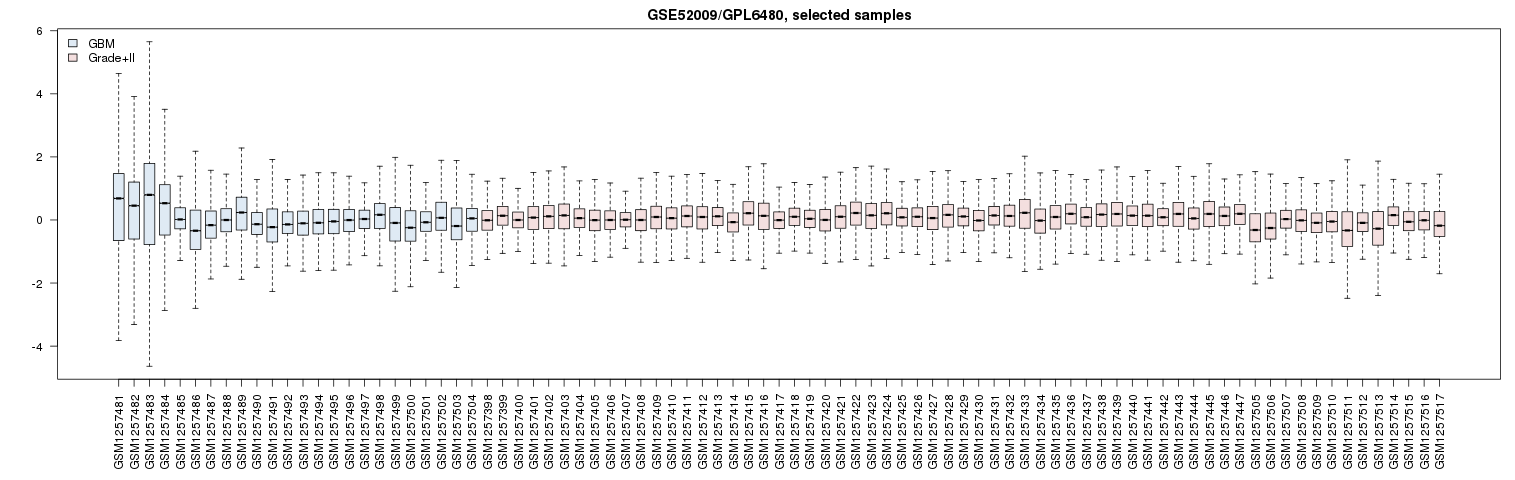

Supplement: Supplementary file 1 [file ijms-19-01369-s001.zip › Supplementary_files/Figure S9 test v2-1.png]

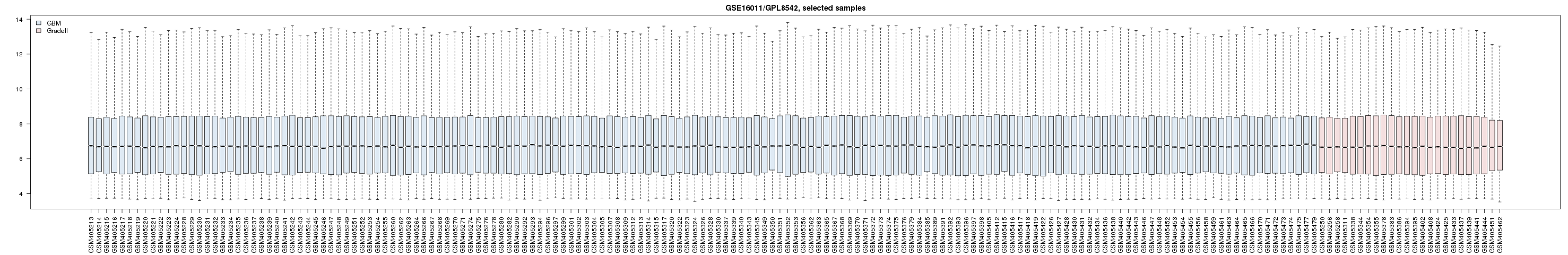

Supplement: Supplementary file 1 [file ijms-19-01369-s001.zip › Supplementary_files/Figure S11 test v2-3.png]

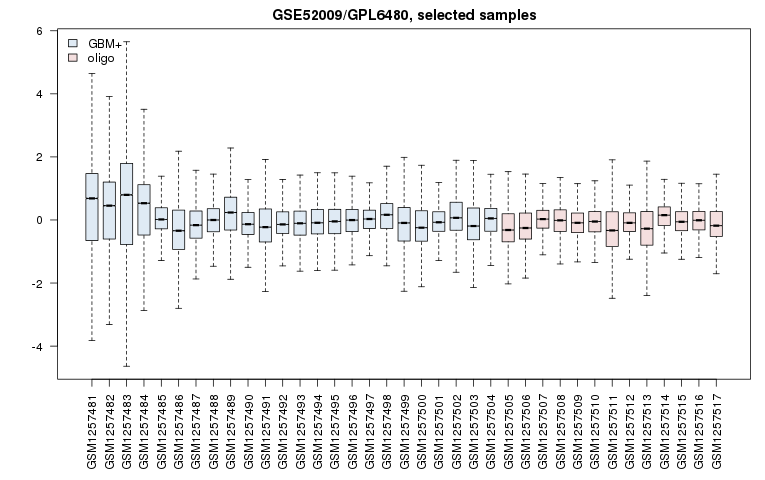

Supplement: Supplementary file 1 [file ijms-19-01369-s001.zip › Supplementary_files/Figure S14 test v3-3.png]

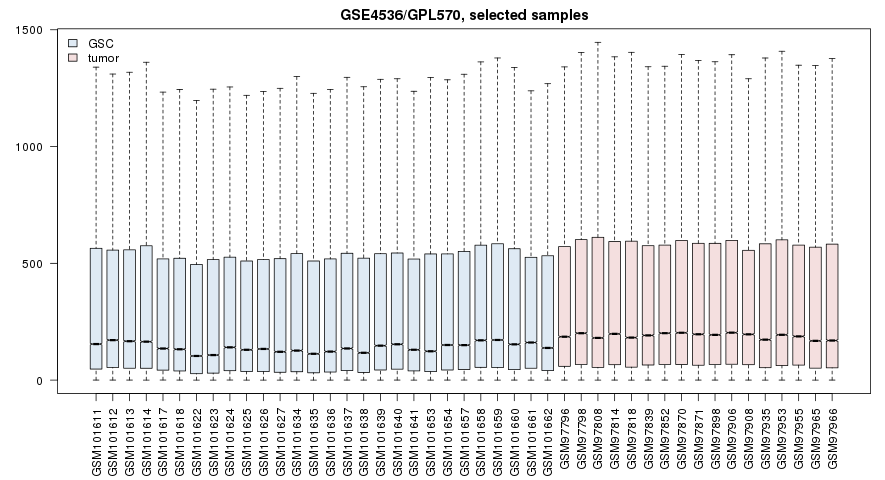

Supplement: Supplementary file 1 [file ijms-19-01369-s001.zip › Supplementary_files/Figure S17 test v4-1.png]

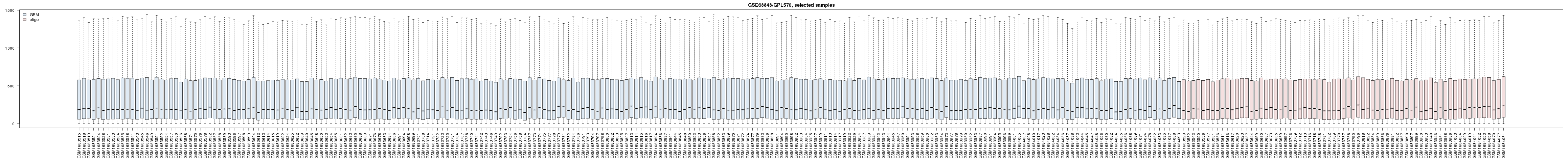

Supplement: Supplementary file 1 [file ijms-19-01369-s001.zip › Supplementary_files/Figure S15 test v3-4.png]

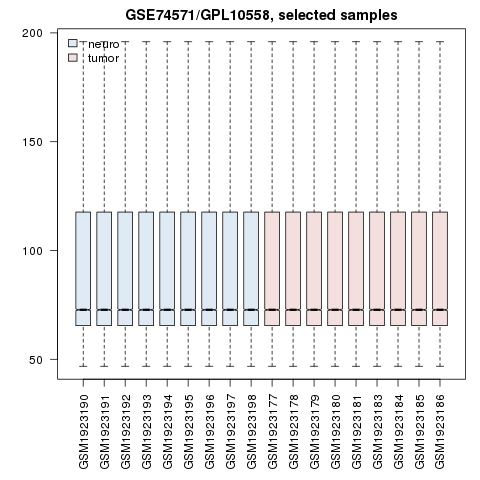

Supplement: Supplementary file 1 [file ijms-19-01369-s001.zip › Supplementary_files/Figure S19 test v6-1.png]

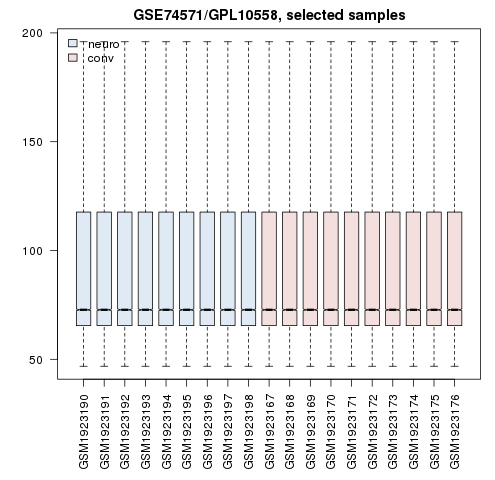

Supplement: Supplementary file 1 [file ijms-19-01369-s001.zip › Supplementary_files/Figure S20 test v7-1.png]
